# Unveiling the diversity of hemoplasmas (hemotropic *Mycoplasma* spp.) in Brazilian wildlife: two decades of molecular discoveries – a scoping review

**DOI:** 10.1590/S1984-29612026007

**Published:** 2026-05-11

**Authors:** Anna Claudia Baumel Mongruel, Lorena Freitas das Neves, João Fabio Soares, Rosangela Zacarias Machado, Rafael Felipe da Costa Vieira, Marcos Rogério André

**Affiliations:** 1 Universidade Estadual Paulista “Júlio de Mesquita Filho” – UNESP, Faculdade de Ciências Agrárias e Veterinárias – FCAV, Departamento de Patologia, Reprodução e Saúde Única, Vector-Borne Bioagents Laboratory – VBBL, Jaboticabal, SP, Brasil; 2 Universidade Federal do Rio Grande do Sul – UFRGS, Faculdade de Veterinária, Departamento de Patologia e Clínica Veterinária, Porto Alegre, RS, Brasil; 3 University of North Carolina at Charlotte, Department of Epidemiology and Community Health, Charlotte, NC, United States of America; 4 University of North Carolina at Charlotte, Center for Computational Intelligence to Predict Health and Environmental Risks – CIPHER, Charlotte, NC, United States of America

**Keywords:** Animals, Brazil, Haemobartonella, hemoparasites, Eperythrozoon, PCR, Animais, Brasil, Haemobartonella, hemoparasitos, Eperythrozoon, PCR

## Abstract

Hemotropic mycoplasmas (hemoplasmas) are epicellular bacteria of the *Mycoplasma* genus capable of attaching to mammalian erythrocytes. Recently, reports of hemoplasmas in Brazilian wildlife have increased exponentially, reflecting advances in molecular diagnosis, bioinformatic analyses, and broader access to these tools. The present scoping review updates the current available information on hemoplasmas occurrence and genetic diversity in Brazilian wildlife, including terrestrial, aquatic, and flying mammals, as well as exotic species. The review included 59 scientific papers published between 2007 and 2025. Most records are concentrated in the Atlantic Forest and Pantanal biomes, with rodents showing the highest genetic diversity, potentially reflected by a larger number of analyzed samples. Some mammal groups harbor apparently host-specific hemoplasmas lineages, such as tapirs (*Tapirus terrestris*), opossums (*Didelphis* spp.), crab-eating raccoons (*Procyon cancrivorus*), and neotropical otters (*Lontra longicaudis*). Collectively, available data demonstrate that hemoplasmas are widespread and frequently reported in Brazilian wildlife. Although the use of multi-locus sequencing and comprehensive phylogenetic reconstructions have provided additional insights when compared to single-gene analyses, their resolution is still limited for clarifying the evolutionary relationships and taxonomic placement of hemoplasmas. Looking ahead, advances in genomic comparisons may further deepen our understanding of hemoplasmas diversity and evolution.

## Resumo

Os micoplasmas hemotrópicos (hemoplasmas) são bactérias epicelulares do gênero *Mycoplasma*, capazes de se aderir aos eritrócitos de mamíferos. Recentemente, os relatos de hemoplasmas em animais silvestres do Brasil aumentaram exponencialmente, refletindo os avanços no diagnóstico molecular, nas análises bioinformáticas e no acesso mais amplo essas ferramentas. A presente revisão de escopo atualiza as informações disponíveis sobre a ocorrência e a diversidade genética de hemoplasmas na fauna silvestre brasileira, incluindo mamíferos terrestres, aquáticos e voadores, bem como espécies exóticas. Foram reunidos 59 artigos científicos publicados entre 2007 a 2025. A maioria dos registros concentra-se nos biomas Mata Atlântica e Pantanal, sendo os roedores o grupo que apresenta a maior diversidade genética, o que está potencialmente relacionado à um maior número de amostras investigadas. Alguns grupos de mamíferos apresentam linhagens de hemoplasmas aparentemente hospedeiro-específicas, como as antas (*Tapirus terrestris*), gambás (*Didelphis spp.*), mão-peladas (*Procyon cancrivorus*) e lontras neotropicais (*Lontra longicaudis*). Coletivamente, os dados disponíveis demonstram que os hemoplasmas são amplamente distribuídos e frequentemente relatados na fauna silvestre brasileira. Embora o uso de sequenciamento *multi-locus* e reconstruções filogenéticas abrangentes tenha fornecido informações adicionais em comparação com análises baseadas em um único gene, sua resolução ainda é limitada para esclarecer as relações evolutivas e o posicionamento taxonômico dos hemoplasmas. No futuro, avanços em comparações genômicas poderão aprofundar ainda mais a compreensão sobre a diversidade e evolução dos hemoplasmas.

## Introduction

### Historical background: from *Eperythrozoon* and *Haemobartonella* to hemoplasmas

Microorganisms of the class Mollicutes are considered the simplest and smallest self-replicating forms of life. These organisms have undergone regressive evolution and genome reduction from more complex ancestors, diverging approximately 65 million years ago (Trachtenberg, 2005), resulting in compact-sized genomes (550-2200 kb), reduced metabolic pathways, and absence of a typical cell wall (Pollack et al., 1997; Rivera-Tapia et al., 2002; Catchpowle et al., 2024).

The lack of a cell wall is a distinguishing characteristic of Mollicutes, and indeed this fundamental characteristic was central to the original proposal of the class name (from Latin: *mollis* = soft; *cutis* = skin) (Edward & Freundt, 1967). In 1965, Tanaka et al. (1965) reported that the erythrocyte-parasite species *Eperythrozoon coccoides* and *Haemobartonella muris*, till then classified as Rickettsiales, do not present a true cell wall, suggesting a separation of these two agents from the rickettsial group. The arguments in favor of this taxonomic separation were further strengthened by studies conducted by Rikihisa et al. (1997) and Neimark & Kocan (1997), which demonstrated a phylogenetic relationship between *Eperythrozoon* spp. and *Haemobartonella* spp. with species from the Mycoplasmataceae family, based on 16S rRNA gene amplification and sequencing. In 2001, the reclassification was officially proposed by Neimark et al., relocating *Eperythrozoon* species to the genus *Mycoplasma*. In addition, the authors pointed out that these species constitute a distinct group of erythrocyte parasitic mycoplasmas and the informal name ‘haemoplasmas’ (or “hemoplasmas”) was proposed (Neimark et al., 2001; Neimark et al., 2002).

Hemoplasmas differ from mucosal *Mycoplasma* species due to their erythrocyte tropism, relatively lower 16S rRNA gene similarity, and failure to grow in vitro (Santos et al., 2011; Descloux et al., 2021). Despite these biological distinctions, hemoplasmas are phylogenetically intermixed among non-hemotropic *Mycoplasma* species. The family *Mycoplasmataceae* exhibits a complex evolutionary structure, and 16S rRNA-based analyses indicate that the genus *Mycoplasma* is polyphyletic, comprising multiple deep lineages. Within this context, hemoplasmas are placed in the so-called “pneumoniae group,” which also includes *Mycoplasma pneumoniae* and related taxa (Brown et al., 2011). Within the hemoplasma group, phylogenetic analyses based on the 16S rRNA gene have further demonstrated the formation of two main clusters: The first includes *Mycoplasma coccoides* together with *Mycoplasma haemocanis* and *Mycoplasma haemofelis*, while the second cluster includes *Mycoplasma ovis*, *Mycoplasma suis,* and *Mycoplasma wenyonii* (Brown et al., 2011). In fact, ‘suis’ and ‘haemofelis’ clusters apparently share the same ancestor but are separated with high support values (Volokhov et al., 2012).

The consequences of hemoplasma infection remain poorly understood in many hosts. Clinical disease has been most frequently described in cats, particularly those infected with *M. haemofelis*, and is characterized by hemolytic anemia and systemic signs. In dogs, *M. haemocanis* infection has been associated with hemolytic crises, especially in splenectomized, immunosuppressed, or co-infected animals (Kemming et al., 2004; Santos et al., 2015). In livestock, infections are typically chronic and may affect productivity and reproductive performance (Tagawa et al., 2013).

The role of ectoparasites as hemoplasma vectors remains unclear for most species. In cats, the transmission of *M. haemofelis* and '*Candidatus* Mycoplasma haematominutum' through *Ctenocephalides felis* fleas was not fully confirmed (Woods et al., 2005, 2006). Among dogs, although the experimental transmission of *M. haemocanis* by *Rhipicephalus sanguineus* sensu lato (s.l.) ticks has been reported (Senevtratna et al., 1973), aggressive interactions seem to play a major role in the transmission of these agents (Huggins et al., 2023). Additionally, the vector competence of *Rhipicephalus microplus* in the transmission of '*Candidatus* Mycoplasma haematobovis' has been reported in mouse models (Shi et al., 2019). In contrast, some studies reported hemoplasma DNA in hosts with no history of ectoparasite infestation (Di Cataldo et al., 2020; Mongruel et al., 2022a; Huggins et al., 2023), and ectoparasites collected from hemoplasma-positive vertebrate hosts do not always test positive for these agents (Sousa et al., 2017).

Transfusion-associated transmission of hemoplasmas has been reported or suggested in dogs and cats (Lester et al., 1995; Nury et al., 2021; Roels et al., 2024). In addition, hemoplasma DNA has been detected in stored feline and canine blood products (Gary et al., 2006; Castillo et al., 2023), with evidence of metabolic activity during storage in canine samples (Castillo et al., 2023), supporting the biological plausibility of transmission through blood transfusion.

Uterine transmission has been described in cows (Girotto-Soares et al., 2016), humans (Yang et al., 2000), dogs (Lashnits et al., 2019), and wild animals (Millán et al., 2024), which may represent an important route in the spread of the agent. In fact, hemoplasmas are believed to use diverse transmission routes, involving arthropod vectors, fomites (iatrogenic), direct transmission via vertical transmission, and, probably, social/aggressive interactions (Millán et al., 2021). Zoonotic infections were reported for *M. suis* (Yuan et al., 2009), *M. haemofelis* (Santos et al., 2008), *M. ovis* variants (Sykes et al., 2010), '*Candidatus* Mycoplasma haematoparvum' (Maggi et al., 2013), and *M. haemocanis* (Kmetiuk et al., 2025a, b). Furthermore, ‘*Candidatus* Mycoplasma haematohominis’, a bacterium widely distributed in bats, is described as a potential cause of severe infections in human beings (Descloux et al., 2021). Such dynamics raise epidemiological concerns in shared habitats where humans and domestic/wild animals coexist, particularly regarding potential zoonotic risks (André et al., 2014).

### General aspects of hemotropic mycoplasmas and their occurrence in Brazil

Hemotropic *Mycoplasma* are epicellular (except for *M. suis* [Groebel et al., 2009]) bacteria that attach to the surface of mammals’ erythrocytes. These bacteria are characterized by a pleomorphic shape and a small circular genome, encoding only genes essential for their survival (Messick, 2004). In Brazil, the main hemoplasma species infecting cats include *M. haemofelis*, ‘*Ca.* M. haematominutum’ and ‘*Candidatus* Mycoplasma turicense’ (Braga et al., 2012; Firmino et al., 2016; Marcondes et al., 2018; Munhoz et al., 2018; Dias et al., 2025). In dogs, *M. haemocanis* and ‘*Ca.* M. haematoparvum’ have been reported (Vieira et al., 2015; Valle et al., 2014; Soares et al., 2016; Miranda et al., 2021).

Regarding farm animals, reports of *M. ovis* infection can be found in sheep (Souza et al., 2019; Mongruel et al., 2020), goats (Machado et al., 2017), and horses (Kakimori et al., 2023), as well as reports of *M. suis* (Guimarães et al., 2007a; Toledo et al., 2016; Gatto et al., 2019) and *Mycoplasma parvum* (Gatto et al., 2019; Sonálio et al., 2021) in pigs. '*Ca.* M. haematobovis' (Girotto et al., 2012; Girotto-Soares, et al., 2016) and *M. wenyonii* have been reported in cattle (De Mello et al., 2019). Regarding the zoonotic transmission, a human patient infected with HIV (human immunodeficiency virus) was coinfected with *M. haemofelis* and *Bartonella henselae* (Santos et al., 2008). Recently, *M*. *haemocanis* was reported in indigenous and quilombolas communities in southern and southeastern Brazil (Kmetiuk et al., 2025a, b).

Moreover, the DNA of different hemoplasma species and genotypes has been detected in potential vectors in the country, such as salivary glands of *Amblyomma dubitatum* (Vieira et al., 2021) and *Dermacentor nitens* ticks (Kakimori et al., 2023), and pools of *Polyplax spinulosa* louse (Gonçalves et al., 2020).

The last review on hemoplasmas occurrence in Brazil was released in 2009 (Biondo et al., 2009). Recently, a comprehensive review included hemoplasmas from wild animals in different parts of the world (Millán et al., 2021). However, no updated review has specifically addressed Brazilian wildlife. Considering the country's vast biodiversity, the advances in molecular investigation, and the extensive reports produced over nearly two decades, the present review aims to provide an updated overview of the occurrence of hemoplasmas in wild hosts in Brazil. Additionally, we aimed to construct a phylogenetic tree based on partial 16S rRNA gene sequences, including most of the hemoplasma sequences reported in Brazilian wildlife that are available in the GenBank database (NCBI, 2025).

## Material and Methods

The present review utilizes the current nomenclature for these microorganisms from the List of Prokaryotic names with Standing in Nomenclature (LPSN). This scoping review was designed following the Preferred Reporting Items for Systematic Reviews and Meta-Analyses extension for Scoping Reviews (PRISMA-ScR) guidelines (Tricco et al., 2018).

Publications were retrieved from PubMed, SciELO, and Google Scholar using the following search string: ("hemoplasma*" OR "hemotropic mycoplasma*" OR "mycoplasma*") AND ("wild*" OR "wildlife") AND “Brazil”. The search encompassed studies published between 2007 and 2025, and only original articles reporting molecular detection or characterization of hemoplasmas in wild mammals from Brazil were included. Special emphasis was placed on the biome and state-level origin of the reports to explore potential eco-epidemiological patterns.

Figures were created using QGIS 3.28 software (QGIS Development Team, 2022), RStudio 4.3.2., and Canva Pro (Canva, 2025).

A phylogenetic tree was constructed aiming to include the maximum number of available sequences. To achieve a robust topology, only partial sequences of the 16S rRNA gene with at least 600 bp were included. A total of 192 homologous hemoplasma sequences deposited in GenBank were aligned using the MAFFT online software (MAFFT, 2025) with default parameters. Two outgroup sequences were included: *Bacillus subtilis* (NR112116) and *M. pneumoniae* (AF132741). The alignment was subsequently edited in BioEdit v.7.2 (Hall, 1999) to reduce variation in sequence length and remove poorly aligned ends (Portik & Wiens, 2021).

Phylogenetic inference was performed using the Maximum Likelihood (ML) method implemented in the online software IQ-TREE (2025). Analyses were conducted under default parameters and ultrafast bootstrap approximation (Minh et al., 2013), with 1,000 bootstrap replicates to assess the robustness of the inferred tree. The best-fit substitution model was selected according to the Akaike Information Criterion (AIC) and implemented in IQ-TREE. Trees were visualized and edited in FigTree v1.3.1 (Rambaut, 2010).

## Results

**Figure 1:**
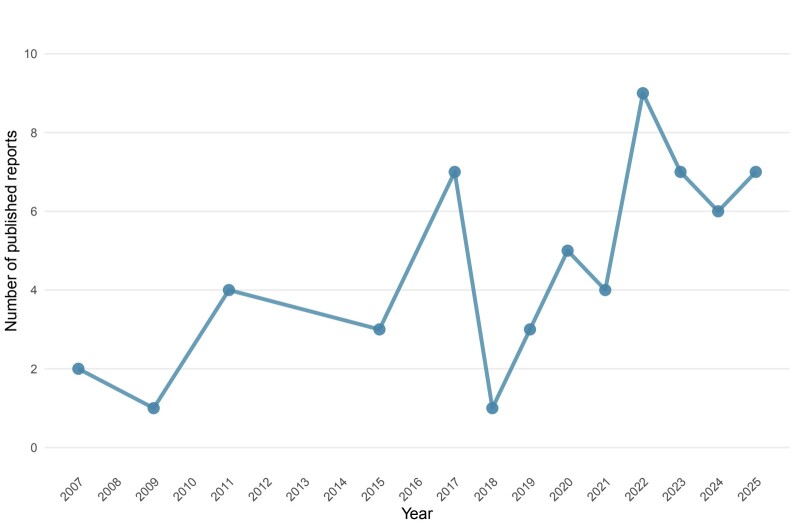
Timeline of publications (2007–2025) reporting the molecular detection of hemoplasmas in wild animals in Brazil included in this scoping review.

The exclusion criteria comprised reports published in conference proceedings, theses, dissertations, revision articles, and studies not focusing on molecular detection of hemoplasmas in Brazilian wildlife. After full-text evaluation, 59 studies met the eligibility criteria and were included in the final analysis. No duplicated data were identified among the included studies. The present review included scientific papers published between 2007 and 2025 (**Figure 1**). The highest number of reports was observed in 2022 (n=9). The overall publication mean during this 19-year period was 3.11 papers per year. However, from 2022 to 2025, the mean annual output was 7.25 papers, reflecting a growing research interest in molecular detection of hemoplasmas in wild animals from Brazil. A summary table describing host species, target gene fragments, geographic origin, and reported clinical signs is provided in **Supplementary File 1** (**Supplementary Material**).

**Figure 2:**
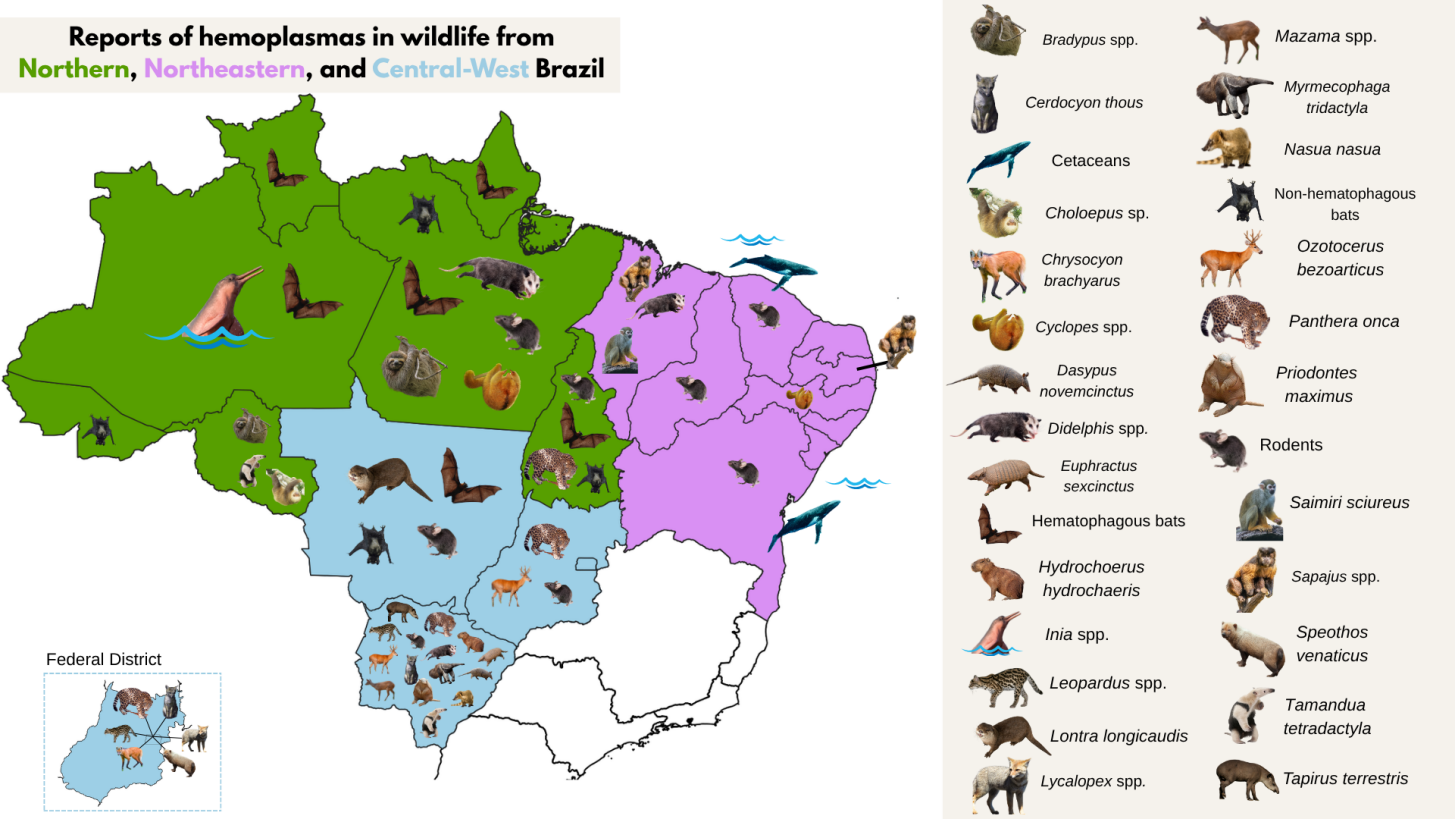
Reports of hemoplasmas in wildlife hosts from Northern, Northeastern, and Central-West Brazil.

**Figure 3:**
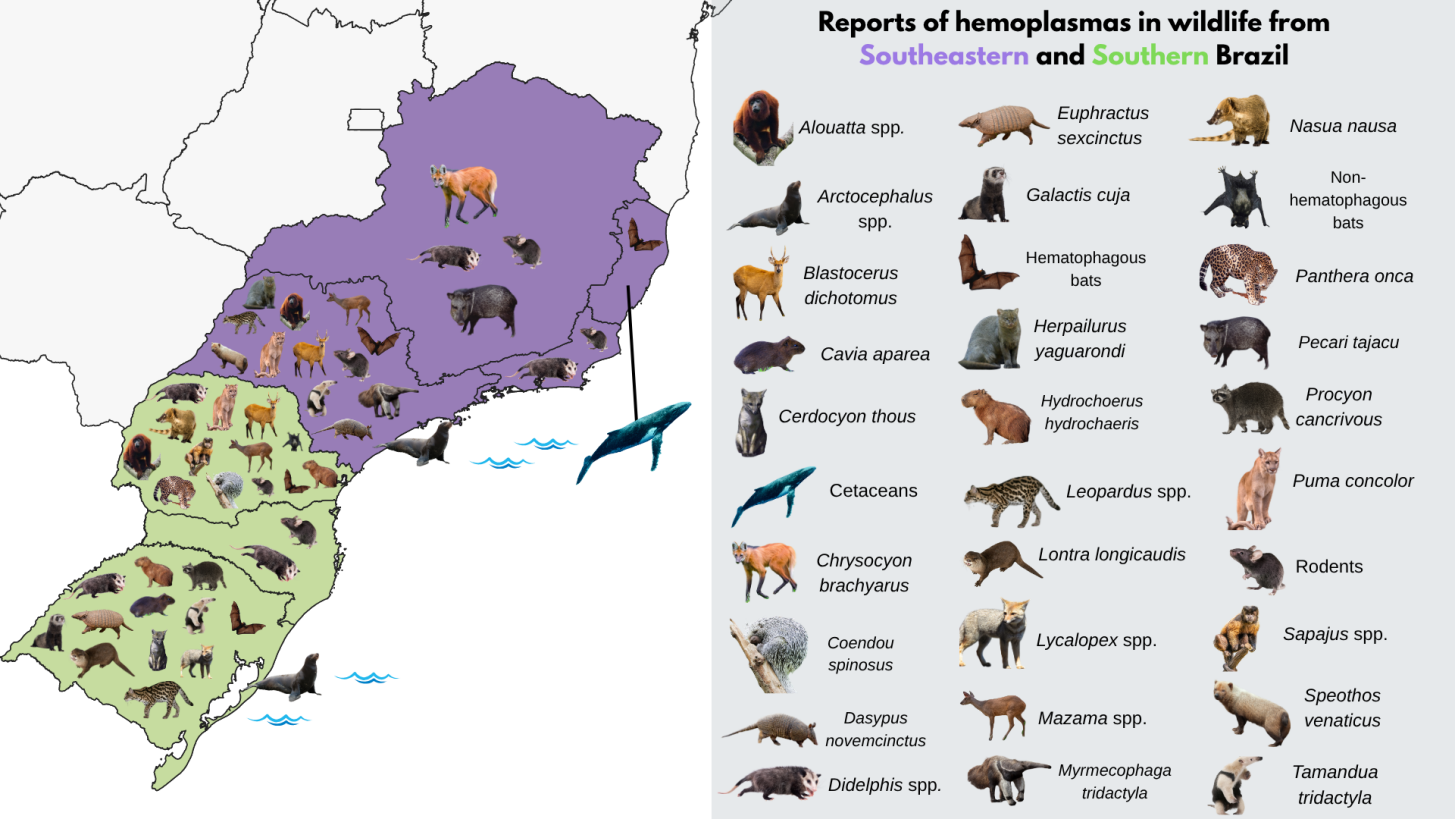
Reports of hemoplasmas in wildlife hosts from Southeastern and Southern Brazil.

Maps were created to illustrate reports of hemoplasmas in different wild hosts from Northern, Northeastern, and Central-West Brazil (**Figure 2**), as well as from Southeastern and Southern Brazil (**Figure 3**). Additionally, a map was created to show reports of hemoplasmas in exotic species (**Supplementary File 2**) and to display the geographical distribution of Brazilian states and biomes (**Supplementary File 3**).

**Figure 4:**
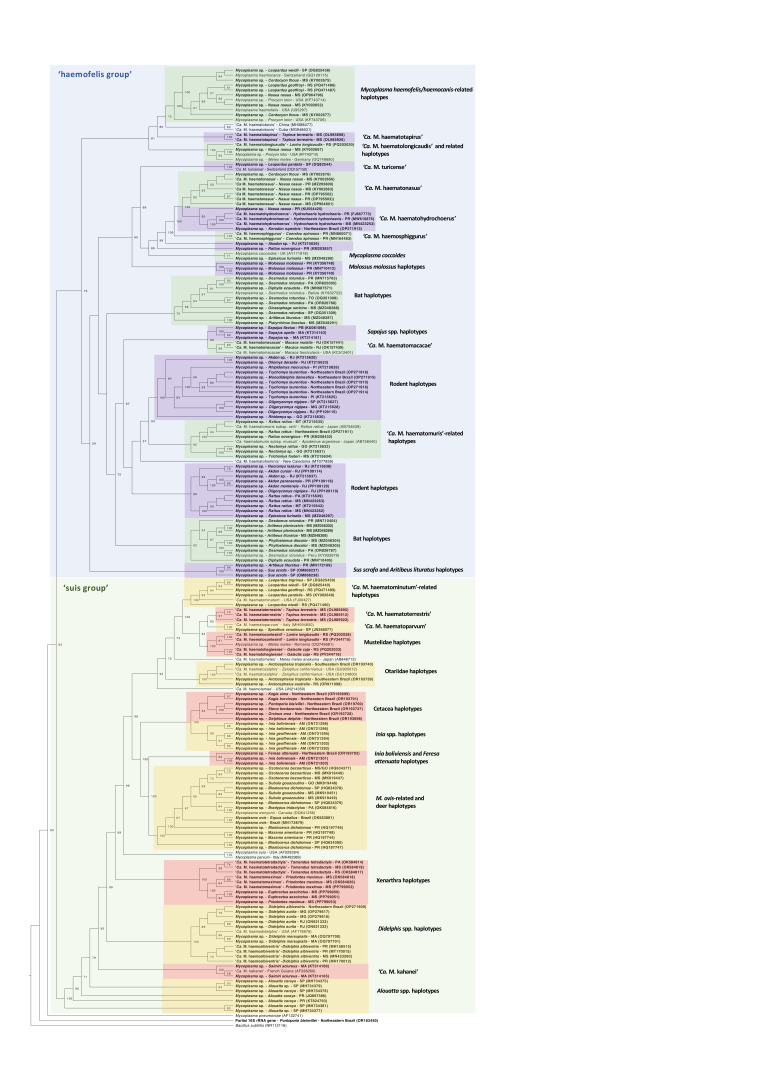
Maximum Likelihood phylogenetic tree based on partial 16S rRNA gene of hemotropic *Mycoplasma* spp. The tree was constructed using an alignment with a total size of 751 bp and GTR+F+G4 substitution model. Sequences obtained from animals in Brazil are highlighted in bold. Sequences of *Mycoplasma pneumoniae* (AF132741) and *Bacillus subtilis* (NR112116) were used as outgroups. Only bootstrap values >50 are shown.

The resulting phylogenetic tree (**Figure 4**) was constructed from a 751 bp alignment, with the GTR+F+G4 model selected as the best-fit substitution model. As expected, two main subgroups were formed: the 'haemofelis group' and the 'suis group', within which different haplotype clusters could be observed. Interestingly, some patterns supported the host–evolutionary relationship hypothesis. For example, hemoplasmas from cetaceans and deer (both belonging to the Artiodactyla order) were positioned closely together. Additionally, considering the large number of sequences included, our analysis revealed interesting insights about the diversity of hemoplasmas in Brazilian wild animals. As an example, sequences obtained from *D. albiventris* from the states of Paraná and Mato Grosso do Sul (‘*Ca*. M. haematoalbiventris’) clustered separately from sequences obtained from *Didelphis* spp. from other geographic regions, which were more closely related to ‘*Ca*. M. haemodidelphis’, reinforcing the occurrence of genetically distinct haplotypes in opossums from Brazil.

Consistent with the literature, bats and rodents showed the greatest genetic diversity, forming distinct clades within the 'haemofelis group'. One sequence obtained from *Pontoporia blainvillei* was positioned outside the main hemoplasma clade, suggesting a possible nonspecific amplification from other bacterial groups. This finding reinforces concerns regarding the specificity of hemoplasma PCR protocols and highlights the importance of sequencing to confirm taxonomic identity.

The main limitation of our analysis lies in the inclusion of short gene fragments (at least 600 bp). However, this criterion was necessary to include the maximum number of available sequences and to assess how they cluster within the tree topology. It is also worth noting that the 16S rRNA gene, although widely used for phylogenetic reconstruction, is highly conserved and therefore allows only haplotype-level differentiation.

## Discussion

### Terrestrial mammals

#### Carnivora

##### Felidae

Hemoplasma infections have been documented globally in both domestic and wild felids, with *M. haemofelis*, ‘*Ca.* M. haematominutum’, and ‘*Ca.* M. turicense’ as the main reported species. *Mycoplasma haemofelis* is typically considered the most pathogenic, often linked to hemolytic anemia in domestic cats, particularly when associated with retroviral infections (Messick, 2004; Sykes & Tasker, 2013). In contrast, wild felids are frequently asymptomatic carriers, although the factors underlying this condition remain unknown. Molecular surveys have shown variable infection rates across species and regions (Willi et al., 2007a). In Brazil, hemoplasma infections in wild felids have been reported across multiple biomes and management conditions, revealing varying prevalence rates and species distribution.

A study performed by Willi et al. (2007a) investigated hemoplasma infections in native and exotic felids in Brazil, most of them maintained in captivity. The study demonstrated that multiple feline hemoplasma species circulate among Brazilian wild felids, including *M. haemofelis*, ‘*Ca*. M. haematominutum’, and ‘*Ca*. M. turicense’. ‘*Ca*. M. haematominutum’ was the most frequently detected agent and was identified in several native species, whereas ‘*Ca*. M. turicense’ was detected only in a free-ranging ocelot (*Leopardus pardalis*). Interestingly, no infections were observed in jaguarundis (*Puma yagouaroundi*) or cougars (*Puma concolor)*, suggesting potential interspecific differences in susceptibility or exposure among felids.

The same study (Willi et al., 2007a) conducted a comprehensive multicontinental investigation on feline hemoplasmas across both free-ranging and captive wild felids, including individuals from Europe, Africa, and South America. Wild felids from Brazil exhibited the lowest hemoplasma prevalence compared to other geographic regions. Moreover, co-infection with multiple hemoplasma species was significantly more frequent in free-ranging animals than in captive individuals, highlighting the potential role of environmental exposure. Notably, significant differences were observed among species regarding overall hemoplasma infection and ‘*Ca*. M. haematominutum’ specifically. For instance, four out of seven ocelots tested positive for ‘*Ca*. M. haematominutum’, despite being housed separately, raising questions about species susceptibility and non-direct transmission routes.

Patterns of co-infection observed in wild felids resemble those described in domestic cats. For instance, ‘*Ca.* M. turicense’ positive cats frequently show co-positivity with ‘*Ca*. M. haematominutum’, reinforcing the hypothesis that these two agents may share common routes of infection or risk factors (Willi et al., 2006, 2007b; Fujihara et al., 2007). Similar co-positivity patterns have also been reported in Brazilian domestic cats, as co-positivity for ‘*Ca*. M. haematominutum’ and *M. haemofelis* is reported in domestic cats (Braga et al., 2012), providing similar infection dynamics as shown in wild felids.

Similarly, ‘*Ca*. M. haematominutum’ was the most frequently detected agent in captive felids from São Paulo. André et al. (2011) detected ‘*Ca*. M. haematominutum’ in different species of captive wild felids (jaguar [*Panthera onca*], cougar*,* ocelots*,* jaguarundi*,* oncilla), whereas *M. haemofelis* was detected in oncillas only; all tested animals were asymptomatic. Later, Ribeiro et al. (2017) reported ‘*Ca*. M. haematominutum’ in asymptomatic, clinically healthy cougars and jaguars at a Paraná zoo. Phylogenetic analysis of the obtained sequences (16S rRNA gene) showed high similarity among the ‘*Ca*. M. haematominutum’ genotypes in these animals. These findings reinforce that these hosts can harbor hemoplasmas silently, which may represent a challenge for the detection and monitoring in zoological settings.

In the Pantanal biome in the Midwest region, State of Mato Grosso do Sul, ‘*Ca*. M. haematominutum’ was detected in free-ranging ocelots (Sousa et al., 2017), corroborating with previous reports of this agent occurring in this felid species (Willi et al., 2007a; André et al., 2011). Later, Carneiro et al. (2020) detected hemoplasma DNA in captive felids from Brasília (Federal District) using 16S rRNA-specific PCR protocols for *M. haemofelis*, ‘*Ca*. M. haematominutum’ (Criado-Fornelio et al., 2003), ‘*Ca.* M. turicense’ (Aquino et al., 2014), *M. haemocanis*, and ‘*Ca.* M. haematoparvum’ (Torkan et al., 2014). Multiple native species were affected (jaguars, ocelots, cougars, and margays), and coinfections were identified, including combinations involving ‘*Ca*. M. haematominutum’ and ‘*Ca*. M. haematoparvum’, as well as detection of typically canine-associated hemoplasmas in wild felids. It is worth noting that 16S rRNA protocols may not be able to differentiate some closely related species, such as *M. haemocanis* and *M. haemofelis*, being necessary to perform additional characterization based on other molecular markers (e.g., RNAse P) (Birkenheuer et al., 2002). Therefore, the hemoplasma species infecting these animals could not be confirmed due to the absence of sequencing.

A study conducted by Furtado et al. (2018) sampled 30 free-living jaguars in three different biomes: Pantanal (Mato Grosso do Sul state), Cerrado (Goiás state), and a transition area between the Amazon and Cerrado (Tocantins state). Once again, ‘*Ca*. M. haematominutum’ was the most frequent species detected in all three biomes. Approximately 30% of the positive animals were co-infected with at least two agents.

In southern Brazil, studies conducted in Rio Grande do Sul have demonstrated frequent detection of hemoplasmas in Geoffroy’s cats and margays from the Pampa and Atlantic Forest biomes (Souza et al., 2025). The species *M. haemofelis* was predominant, followed by ‘*Ca.* M. turicense’ and ‘*Ca*. M. haematominutum’, and cases of triple infection were also reported. The authors suggested that the higher occurrence of *M. haemofelis* found may be attributed to the eco-epidemiological complexity of hemoplasmas, potentially due to exposure to vectors, a naturally higher presence of this agent in the Pampa biome, and factors related to host ecology and susceptibility. Factors such as species, sex, age group, pelage, capture season, and the presence of ectoparasites did not present statistical significance. Similarly, Lignon et al. (2025) reported hemoplasma DNA in the carcasses of Geoffroy's cats from the same region, showing 98.34% similarity to ‘*Ca*. M. haematominutum’ previously isolated from a domestic cat. Together, these findings suggest that southern Brazil may represent a region of intense hemoplasma circulation among small wild felids.

Previous studies indicated that wild felids may serve as long-term reservoirs of hemoplasmas in natural ecosystems. Hemoplasma DNA was reported persistently or transiently in Geoffroy's cat individuals sampled over 24 months, reinforcing the idea of chronic or fluctuating bacteremia and their potential as reservoirs. Recaptured individuals presented positive results, mainly for *M*. *haemofelis*, demonstrating maintenance of infection or reinfection and their potential role as reservoirs (Souza et al., 2025). Similar infection patterns have been observed with *M. haemofelis* and ‘*Ca*. M. haematominutum’ in domestic cats, while ‘*Ca.* M. turicense’ typically exhibits lower parasitemia detectable only through sensitive molecular assays (Tasker, 2010; Di Cataldo et al., 2020).

Across previous studies, hemoplasma infections in wild felids in Brazil appear to be common (prevalence ranging from 13% to 73%), although they have been considered less frequent than in other countries. When not co-occurring with other agents, hemoplasma infections are predominantly subclinical for both free-ranging and captive felids. This is particularly evident in zoo-housed animals, which are regularly monitored and yet rarely show clinical signs suggestive of acute infection (Ribeiro et al., 2017; André et al., 2011; Carneiro et al., 2020). Most infected animals did not exhibit clinical or hematological abnormalities, reinforcing the notion that hemoplasma infections in wild felids may often be subclinical (Carneiro et al., 2020). In contrast, Furtado et al. (2018) reported an adult female jaguar from the Amazon region that was underweight and dehydrated; however, this animal was co-infected with hemoplasmas, *Hepatozoon* spp., and *Cytauxzoon felis*. The absence of clinical signs in single-agent infections, together with evidence of chronic infections, suggests possible host adaptation or infection latency mechanisms.

##### Canidae

Molecular studies across different biomes and management conditions in the country have revealed a broader host range and highlighted the ecological relevance of wild canids as potential reservoirs of hemoplasmas. André et al. (2011) reported hemoplasma infection in captive wild canids from zoological institutions in São Paulo, Mato Grosso, and the Federal District. Interestingly, bush dogs (*Speothos venaticus*) tested positive for a hemotropic *Mycoplasma* sp. closely related to ‘*Ca*. M. haematoparvum’ from Mediterranean dogs.

Later, Sousa et al. (2017) detected sequences with 100% of identity with in *M. haemocanis* free-ranging crab-eating foxes (*Cerdocyon thous*) in the Pantanal biome, Mato Grosso do Sul. Phylogenetic analysis of the three *C. thous*-associated sequences revealed distinct clustering, suggesting that crab-eating foxes in the Pantanal may be susceptible to infection by different hemoplasma genotypes. The frequent identification of genetically similar hemoplasmas among different host species supports the idea that some genotypes may lack strict host specificity (Millán et al., 2021).

Carneiro et al. (2020) evaluated hemoplasma infection in captive wild canids in Brasília, Federal District. A prevalence of 83% was reported and positive individuals included maned wolves (*Chrysocyon brachyurus*), hoary foxes (*Lycalopex vetulus*), crab-eating foxes, and two bush dogs. Since sequencing was not performed for the positive samples, it was not possible to confirm the species of hemoplasma infecting the animals. The authors suggest that local environmental factors, such as proximity among enclosures and the presence of synanthropic animals, may facilitate pathogen transmission.

Additional support for hemoplasma exposure in wild canids was reported by Castillo et al. (2024), which has described hemoplasma infection in a maned wolf in a zoo in Minas Gerais state. However, the lack of DNA sequencing precluded species or genotype assessment. Moreover, in Rio Grande do Sul (Pampa biome), Lignon et al. (2025) detected *Mycoplasma* spp. DNA in carcasses of crab-eating foxes and one pampas fox (*Lycalopex gymnocercus*). Phylogenetic analysis showed that the obtained sequences clustered with hemotropic *Mycoplasma* sp. detected in six-banded armadillos (*Euphractus sexcinctus*) within the same study. However, the limited number of taxa included in the phylogenetic analysis precludes broader conclusions.

In general, the occurrence of hemoplasma in wild canids may vary, with prevalence ranging from 4% to 83%. Moreover, the consistent detection of *M*. *haemocanis* and ‘*Ca*. M. haematoparvum’ across different hosts and regions suggests that these pathogens may be silently maintained within native canid populations. Environmental factors, such as enclosure proximity, contact with domestic or synanthropic animals, and aggressive interactions, appear to play important roles in transmission dynamics.

Although the infection of hemoplasmas in wild canids is often linked to the absence of clinical or hematological manifestations (André et al., 2011; Carneiro et al., 2020), coinfections may exacerbate clinical manifestations, particularly in young or immunocompromised individuals. This potential influence was observed in a maned wolf coinfected with *Anaplasma platys* and hemoplasma; and one hoary fox with triple infection including *Ehrlichia canis*, *A. platys,* and hemoplasmas (Carneiro et al., 2020). Both wild canids developed thrombocytopenia, and the maned wolf also presented anemia.

##### Mustelidae

Recently, Baggio-Souza et al. (2025) reported the first detection of hemoplasmas in Brazilian mustelids. Blood and tissue samples from neotropical otters (*Lontra longicaudis*) and lesser grisons (*Galictis cuja*) sampled in the South and Central-West regions of Brazil were screened and most positive cases originated from Southern Brazil. The study described three novel putative species, named *‘Candidatus* Mycoplasma haematocontesinii’, *‘Candidatus* Mycoplasma haematolongicaudis*’*, and *‘Candidatus* Mycoplasma haematohagiwarae’.

Sequences of ‘*Ca.* M. haematocontesinii’ were obtained from *L. longicaudis* sampled in Atlantic Forest areas of Rio Grande do Sul, clustering closely with ‘*Ca. M.* haematohagiwarae’, which was detected in *G. cuja* from Pampa areas in the same state. Both were grouped within the "suis group," closely positioned to a genotype previously identified in European badger (*Meles meles*) in Europe. In contrast, ‘*Ca.* M. haematolongicaudis’ sequences, recovered from *L. longicaudis* in both Atlantic Forest and Pampa areas of Rio Grande do Sul, were positioned within the "haemofelis group," alongside hemoplasmas previously reported in European badger in Europe and raccoon (*Procyon lotor*) from the USA. Notably, phylogenetically distinct genotypes were detected within the same host species and geographic areas, reinforcing previous reports of hemoplasmas in Brazilian wildlife, such as in tapirs (*Tapirus terrestris*) (Mongruel et al., 2022b). There is no information available on clinical or hematological changes in hemoplasma-infected mustelids.

##### Procyonidae

Procyonids are plantigrade carnivores belonging to the family Procyonidae. Four species of procyonids can be found in Brazil: Brazilian raccoon (*Procyon cancrivorus*), coatis (*Nasua nasua*), janau (*Bassaricyon alleni*)*,* and jupara (*Potos flavus*). Sousa et al. (2017) demonstrated high occurrence (77%) of hemoplasmas in wild coatis from the Pantanal (Mato Grosso do Sul). Obtained sequences showed relatedness to *Mycoplasma* sp. detected in raccoons in the United States. Phylogenetic and haplotype analyses revealed two main clusters, both positioned into the ‘haemofelis group’: the first included coati sequences grouped with *M. haemocanis* and *M. haemofelis*, along with hemoplasmas from domestic dogs and *C. thous* sampled in the same study. The second cluster consisted of sequences from coatis, and one from *C. thous*, which formed a separate and highly supported clade, phylogenetically close to the genotype described in capybaras and ‘*Ca*. Mycoplasma turicense’, all sharing a single haplotype. These findings suggested the circulation of novel hemoplasma genotypes among coatis in the Pantanal. The authors suggested that the high prevalence observed is possibly linked with the coatis' gregarious behavior.

Similarly, Cubilla et al. (2017a) reported hemoplasma infection in captive coatis from southern Brazil (Paraná State, within the Atlantic Forest). Fragments of the 16S rRNA gene with different base pair sizes presented relatedness with *M. haemofelis* and hemoplasmas previously detected in capybaras from Brazil (which was later identified as ‘*Candidatus* Mycoplasma haematohydrochoerus’).

Later, Collere et al. (2021) described the occurrence of hemoplasmas in wild coatis from the Iguaçu National Park (Paraná state, Atlantic Forest). The obtained sequences were phylogenetically positioned alongside those previously described in coatis from Mato Grosso do Sul (Sousa et al., 2017) and Paraná (Cubilla et al., 2017b). Based on analyses using partial 16S rRNA and 23S rRNA genes, the authors proposed the putative name ‘*Candidatus* Mycoplasma haematonasua’ for a potential new hemoplasma species occurring in coatis in Pantanal (central-western) and Atlantic Forest (southern).

Perles et al. (2023a) reported a remarkably high prevalence of hemoplasmas in coatis from urban areas of Mato Grosso do Sul. Two distinct genotypes were identified and differentiated based on melting temperature: myc1, which predominated among positive samples, and myc2, detected less frequently. Phylogenetic analysis showed that myc1 clustered in the same clade as ‘*Ca.* M. haematonasua’, previously described in coatis from the Pantanal and Atlantic Forest (Sousa et al., 2017; Cubilla et al., 2017b; Collere et al., 2021); while myc2 clustered with raccoon-associated hemoplasmas and *M. haemofelis*. Detection of hemoplasma DNA in pools of *Amblyomma* spp., *Amblyomma sculptum*, and *A. dubitatum* ticks collected from infected coatis, exclusively corresponding to the predominant genotype, was reported. Fluctuation in the estimated bacterial load was observed between different samplings in recaptured animals, consistent with chronic infection patterns. Evidence of coinfection between the two genotypes or hematological alterations was not reported.

Similarly, Perles et al. (2023b) reported a high prevalence (85.7%) of coatis positive for hemoplasmas in the Iguaçu National Park, southern Brazil, in an area of Atlantic Forest. Obtained sequences showed 99.8–100% identity and were phylogenetically positioned together with ‘*Ca*. Mycoplasma haematonasua’. The same study also reported the occurrence of *Anaplasma* spp., *Bartonella* spp., and *Hepatozoon* spp. in the coatis, with hemoplasmas being the most prevalent agents detected in the population.

Regarding other procyonid species, few reports are available. Fagundes-Moreira et al. (2023) described the occurrence of hemoplasmas in Brazilian raccoons. Blood and spleen samples were obtained from raccoons sampled in Pampa (Rio Grande do Sul), Atlantic Forest (Santa Catarina and Paraná), and in the floristic tension zone between the two biomes. The 16S rRNA gene analyses revealed the occurrence of at least three distinct genotypes: two genotypes detected in the Pampa and Atlantic Forest were related to previously described genotypes from raccoons in the USA; the third one, observed in the floristic tension zone, was phylogenetically related to ‘*Ca*. M. haematominutum’. These results indicate a considerable genetic diversity of hemoplasmas in *P. cancrivorus* from southern Brazil.

In summary, the detection of hemoplasmas in procyonids from Brazil has mainly focused on coatis. Indeed, hemoplasmas appear as the most prevalent blood-borne agents in these hosts. Studies conducted in different regions of Brazil demonstrate a high prevalence of hemoplasmas in coatis, ranging from 44.4% to 88.6% in populations sampled in the Atlantic Forest and Pantanal. Phylogenetic analyses based on the 16S rRNA gene revealed the occurrence of at least two genotypes, both positioned into the ‘haemofelis group’: I. Genotypes related to *M. haemofelis*, which are less frequently reported; and II. A cohesive clade comprising sequences identified as '*Ca*. Mycoplasma haematonasua', a potential new species reported in the Pantanal and Atlantic Forest biomes, suggesting a widespread circulation of this genotype among coati populations from different regions in Brazil.

Light microscopy of stained blood smears of hemoplasma-positive coatis revealed small coccoid basophilic epi-erythrocytic structures, often occurring singly or in multiple inclusions per erythrocyte (Cubilla et al., 2017a). Similarly, Giemsa-stained blood smears from a positive raccoon showed basophilic coccoid structures adhered to red blood cells compatible with hemoplasma inclusions (Fagundes-Moreira et al., 2023). The absence of significant hematological alterations in positive animals, together with the high prevalence observed, suggests that these infections have chronic and subclinical presentations, and are possibly favored by coatis' gregarious social behavior.

#### Primates

Hemoplasmas reports concentrate in three native NHP groups in Brazil: howler-monkeys (*Alouatta* spp.) (Cubilla et al., 2017b; Melo et al., 2019), capuchin-monkeys (*Sapajus* spp.) (Bonato et al., 2015; Ramalho et al., 2017; Cubilla et al., 2017b; Melo et al., 2019), and squirrel-monkeys (*Saimiri sciureus*) (Bonato et al., 2015).

In the Brazilian Amazon (Maranhão state), hemoplasma DNA was detected in capuchin-monkeys, black marmosets (*Saguinus midas niger*) and squirrel-monkeys. No individuals of night monkeys (*Aotus infulatus*) or white-tufted marmosets (*Callithrix jacchus*) were positive. Although detection rates varied among host species, interpretation for some taxa is limited by small sample sizes. Phylogenetic analyses revealed two distinct lineages: Sequences from capuchin monkeys clustered as a sister clade to ‘*Candidatus* Mycoplasma haematomacaque’, previously reported in *Macaca* spp. from Japan and the United States, within the “haemofelis group.” In contrast, sequences obtained from squirrel-monkeys grouped with ‘*Candidatus* Mycoplasma kahanei’ identified in the same host species in the United States, suggesting that *S. sciureus* may represent a natural host for this hemoplasma species. The two positive samples from black marmosets did not yield sequences with satisfactory quality, and their phylogenetic positioning could not be evaluated (Bonato et al., 2015).

Cubilla et al. (2017b) reported the occurrence of hemoplasmas in captive and free-range NHP from southern Brazil, within an Atlantic Forest area (Paraná State). Black howler monkeys (*Alouatta caraya*) were significantly more positive than black-horned capuchins (*Sapajus nigritus*). Moreover, captive animals that were born wild were more likely to test hemoplasma-positive than captive-born animals. In phylogenetic analysis, hemoplasma sequences from black howler monkeys were positioned within the ‘suis group’, forming a clade close to *‘Ca.* M. kahanei’, albeit separated with high bootstrap support. Representatives of white-tufted were sampled and yielded negative results, similar to the results found by Bonato et al. (2015).

Ramalho et al. (2017) investigated the presence of hemoplasmas in Marcgrave's capuchin monkeys (*Sapajus flavius*), a critically endangered species, kept in captivity in an area that covers Atlantic Forest and Caatinga biomes (state of Paraíba). Sequences detected among these animals presented 99% of identity with hemotropic *Mycoplasma* sp. previously identified in *Sapajus* spp. from the Amazon biome (Bonato et al., 2015), in addition to 95% of similarity to ‘*Ca*. M. haematomacaque’ reported from Japan and the United States. Phylogenetic analysis placed the hemoplasma obtained sequences in the ‘haemofelis group’, forming a clade with hemoplasma sequences from *Sapajus* spp.

Melo et al. (2019) analyzed the occurrence of hemoplasmas in howler monkeys kept in captivity in the Atlantic Forest region (São Paulo State) and detection rates were lower than those previously described in black howler monkeys from southern Brazil (Cubilla et al., 2017b). Phylogenetic analyses showed that the obtained 16S rRNA gene sequences were genetically close to '*Ca*. M. kahanei', previously detected in *S. sciureus* from Brazil (Bonato et al., 2015), although positioned in distinct clades. In fact, sequences obtained from howler monkeys formed three closely related clusters, all within the suis group. Notably, a sequence previously reported in black howler monkeys from southern Brazil (Cubilla et al., 2017b) also clustered within one of these clusters, suggesting circulation of related hemoplasma genotypes among howler populations across different regions.

In summary, studies conducted to date in native NHP from Brazil revealed the occurrence of at least three major hemoplasma phylogenetic groups: I. The *Sapajus*-related genotypes, within the ‘haemofelis group’, representing hemoplasmas detected in *Sapajus* spp. from the Amazon, Atlantic Forest, and Cerrado biomes, in northeastern Brazil; II. The *Alouatta*-related genotypes, within the ‘suis group’, which includes genetically diverse sequences identified in *Alouatta* spp. from the Atlantic Forest biome in southeastern and southern Brazil; and III. The *S. sciureus*-related group, possibly ‘*Ca*. M. kahanei’, also within the ‘suis group', was found in *S. sciureus* in an Amazon area from northeastern Brazil.

In general, hemoplasma infections in wildlife appear to follow a subclinical course or exhibit low pathogenicity. However, some studies have reported hematological alterations in infected animals. For instance, Cubilla et al. (2017b) observed increased total plasma protein and mean corpuscular volume in non-human primates, suggesting inflammation and macrocytosis without a clear association with anemia. Similarly, Melo et al. (2019) reported significantly higher monocyte and lymphocyte counts, together with decreased platelet levels, in hemoplasma-positive howler monkeys.

#### Artiodactyla

Molecular investigations across different Brazilian biomes have demonstrated a consistent occurrence of *M. ovis* and related hemoplasma lineages in both free-ranging and captive deer populations. Grazziotin et al. (2011a) described the first report of hemoplasma detection in deer from Brazil. Positive pigmy brockets (*Mazama nana*), red brockets (*Mazama americana*), and marsh deer (*Blastocerus dichotomus*), were kept in captivity in the state of Paraná (Atlantic Forest biome). Most isolates shared high sequence identity (98.2–98.4%) with *M. ovis*, suggesting the circulation of variant strains within the captive group. However, one isolate exhibited only 94.9% identity to the reference sequence, raising the possibility of a divergent lineage or even a novel hemoplasma taxon. In fact, the obtained sequences presented divergent phylogenetic positions: most clustered in a clade closest to an *M. ovis*-like sequence previously detected in white-tailed deer (*Odocoileus virginianus*) in the United States (Genbank access: FJ824847). However, the hemoplasma sequence obtained from marsh deer presented a more divergent positioning outside this group. The study highlights the occurrence of at least one potentially new genotype in deer from Brazil and suggested that the high prevalence observed may reflect the ease of transmission of hemoplasmas between individuals of this order.

In the same year, the same research group reported the occurrence of hemoplasmas in wild deer from different regions of the country (Grazziotin et al., 2011b). Samples were obtained from marsh deer collected in flooded areas of São Paulo state, and pampas deer (*Ozotoceros bezoarticus*) captured in the Pantanal of Mato Grosso do Sul, and in the Cerrado biome in Goiás State. Phylogenetic analyses revealed three distinct genotypes: the first, most frequently detected, formed a sister clade to *M. ovis* sequences, clustering with a sequence previously described in white-tailed deer in the United States; the second clustered with ‘*Candidatus* Mycoplasma erythrocervae’, in a clade outside the *M. ovis* clustering, sharing a common ancestor with *M. wenyonii*; and the third, most divergent, clustered with a sequence previously obtained in pampas deer in a study with captive deer (Grazziotin et al., 2011a). The study also highlighted a higher prevalence of hemoplasmas in more humid regions (particularly in the Pantanal and other seasonally flooded ecosystems), compared to the Cerrado biome, suggesting a possible influence of environmental factors on the transmission dynamics of hemoplasmas. Those findings suggest a potential spatial distribution pattern, where ecological variables may influence hemoplasma diversity.

André et al. (2020) reported hemoplasma infections in wild cervids sampled across four Brazilian states (Mato Grosso do Sul, São Paulo, Goiás, and Paraná). Positive cases were identified in gray brockets (*Subulo gouazoubira*), pigmy brockets*,* red brockets, and pampas deer. Phylogenetic analyses revealed that all obtained sequences clustered with others previously detected in deer from Brazil and other regions of the world, forming a distinct clade with high support, separate from the *M. ovis* lineage associated with humans, sheep, and goats. Findings from this study indicated the existence of deer-specific *M. ovis* lineages.

In general, the main hemoplasma genotypes detected in deer from Brazil are genetically close to, albeit distinct from, *M. ovis* detected in domestic animals and humans. All sequences reported from deer in Brazil were positioned within the ‘suis group'. To date, no evidence of pathogenicity associated with infection in these hosts has been observed (Grazziotin et al., 2011b).

Peccaries are members of the Artiodactyla order and Tayassuidae family. Although white-lipped (*Tayassu pecari*) and collared peccaries (*Pecari tajacu*) occur sympatrically, the two species differ in several aspects, such as group size, movement patterns, morphology, and habitat use (Azevedo & Conforti, 2008). A study that evaluated captive peccaries (*P. tajacu* and *T. pecari*) in the state of Paraná (Atlantic Forest) reported the absence of hemoplasmas (Vieira et al., 2011). Similarly, Dias et al. (2019) also reported negative results in 100 individuals of *T. pecari* kept in captivity on a conservation farm located on the border between the states of Minas Gerais, Goiás, and Bahia, using a qPCR protocol specific for *M. suis/M. parvum*.

On the other hand, Silveira et al. (2024) reported positivity in captive *P. tajacu* in the state of Minas Gerais, in the Atlantic Forest area, using a PCR protocol (Criado-Fornelio et al., 2003) capable of detecting multiple hemoplasma species. However, the study did not report the sequencing of the amplicons obtained, which limits further investigation of the genotypes involved. These findings suggest that peccaries from Atlantic Forest areas may harbor hemoplasma genotypes distinct from those typically described in domestic pigs (*M. suis* and *M. parvum*).

#### Perissodactyla

The lowland tapir (*Tapirus terrestris*) is the wild representative of the Peryssodactyla order and the largest land mammal in Brazil. Wild tapirs from Pantanal and Cerrado biomes were positive for hemoplasmas. Most of the positive tapirs were sampled in the Pantanal region and two distinct *Candidatus* species were reported in these animals: I. '*Candidatus* Mycoplasma haematoterrestris', found in animals from both the Pantanal and Cerrado, and phylogenetically positioned within the ‘suis group’; II. '*Candidatus* Mycoplasma haematotapirus', found in animals from the Pantanal and positioned within the ‘haemofelis group' (Mongruel et al., 2022b). The phylogenetic analysis of the 16S rRNA gene sequence obtained from a single tapir demonstrated the presence of '*Ca*. M. haematoterrestris', while the phylogenetic analysis of the sequence obtained through amplification of the 23S rRNA gene indicated the occurrence of '*Ca*. M. haematotapirus', suggesting a possible co-infection with both *Candidatus* species in the same host. Furthermore, some animals tested positive for *'Ca.* M. haematoterrestris' in samples collected on different dates (with an average interval of six months), suggesting the occurrence of chronically infected animals or reinfection events (Mongruel et al., 2022b). There is no information available on clinical or hematological changes in hemoplasma-infected tapirs.

#### Rodentia

A nationwide molecular survey conducted by Gonçalves et al. (2015) evaluated 500 wild rodents’ samples across five Brazilian biomes (Atlantic Forest, Amazon Forest, Caatinga, Cerrado, and Pantanal). An overall hemoplasma prevalence of 21.9% was obtained and positive rodents belonged to *Akodon* spp., *Necromys lasiurus*, *Delomys dorsalis*, *Oligoryzomys* spp., *Oxymycterus* sp., *Trichomys* spp., *Calomys* spp., *Rattus rattus*, *Rhipidomys* spp., *Nectomys* spp., *Hylaemys* sp., *Mus musculus*, and *Oecomys* sp. species. Prevalence varied across regions, being lowest in the Caatinga (9.3%) and highest in the Atlantic Forest (26.2%), suggesting that ecological and climatic factors may influence the circulation of these agents. Phylogenetic analysis revealed eight distinct clusters distributed across different rodent hosts and biomes, all positioned within the *M. haemofelis* group, demonstrating high genetic diversity of rodent hemoplasmas in Brazil. In the southern Pantanal region of central-western Brazil, Sousa et al. (2017) detected hemoplasma DNA in one *Oecomys mamorae*. The sequence clustered within the haemofelis group and showed close relatedness to *M. haemomuris*, previously reported in Pantanal rodents (Gonçalves et al., 2015). The findings suggest that *M. haemomuris* may represent the predominant hemoplasma species infecting wild rodents in this biome.

In Paraná State, 22% of orange-spined hairy dwarf porcupines (*Coendou spinosus*) were positive for hemotropic *Mycoplasma* spp. (Valente et al., 2020). Most porcupines were parasitized by ticks (mainly *Amblyomma longirostre*). Sequences of partial 16S rRNA showed 97% identity with strains previously detected in crab-eating foxes and coatis from the Pantanal (Sousa et al., 2017), but phylogenetically formed a distinct, well-supported clade within the haemofelis group. Partial 23S rRNA sequences also clustered separately and shared only ~82% identity with *M. haemocanis* and *M. haemofelis*. These divergence rates may indicate a genetically distinct evolutionary lineage, possibly linked to the arboreal and forest-dwelling behaviour of *C. spinosus*, which contrasts with the more terrestrial habits of other mammal species, potentially limiting transmission routes. Based on these aspects, a *Candidatus* species was proposed, namely ‘*Candidatus* Mycoplasma haemosphiggurus'.

Hemoplasmas were first reported in capybaras (*Hydrochoerus hydrochaeris*) in Paraná State. Overall, 64% of the animals tested positive and a significantly higher prevalence was observed in free-ranging when compared to captive animals (Vieira et al., 2009). Nearly complete 16S rRNA sequences indicated a potentially novel genotype, sharing 92% identity with *M. coccoides* and 86% with *M. haemomuris*, while all samples were negative for *M. haemomuris* (Vieira et al., 2009). Subsequently, in the Pantanal biome, Gonçalves et al. (2020) reported the occurrence of hemoplasmas in 50% of the sampled capybaras. Most animals were infested with ticks (*A. dubitatum, A. sculptum*, and *Amblyomma* sp.), and the DNA sample from one female *A*. *sculptum* was positive for hemoplasma. Sequences showed 100% identity with a *Mycoplasma* sp. previously reported in capybaras from southern Brazil (Vieira et al., 2009), and clustered in a well-supported clade, reinforcing the occurrence of a capybara-related genotype and being detected in different regions.

Another study with capybaras conducted in Paraná State revealed a high prevalence (94%) of hemoplasma in free-ranging animals. All capybaras were tick-infested at the time of sampling, and hemoplasma DNA was detected in the salivary glands of one tick (*A. dubitatum*), supporting the potential involvement of ectoparasites in transmission. The amplified fragments showed 99–100% identity to capybara-related genotypes previously reported in Brazil (Vieira et al., 2009; Gonçalves et al., 2020). Interestingly, *M. coccoides*-specific PCR, fewer samples tested positive, indicating that species-specific assays may underestimate hemoplasma prevalence in wildlife (Millán et al., 2021). Phylogenetically, 16S rRNA sequences clustered near *Mycoplasma* spp. from *N. nasua* and *C. thous* in the Pantanal, while 23S rRNA sequences formed a sister clade to ‘*Ca*. M. haemosphiggurus’. These findings supported the proposal of a novel species, ‘*Candidatus* Mycoplasma haematohydrochaeris’ (Vieira et al., 2021). Later, a molecular survey from the Pelotas microregion, within the Pampa biome, reported one guinea pig (*Cavia aperea*) and two capybaras positive for *Mycoplasma* sp. (Lignon et al., 2025). Phylogenetic analyses reinforced the positioning of capybara-related hemoplasmas close to ‘*Ca*. M. haematonasua’ and *Mycoplasma* sp. from other rodents.

In the Caatinga biome (Northeastern Brazil), Torres-Santos et al. (2024) reported an overall prevalence of 16.2% for the occurrence of hemotropic *Mycoplasma* in rodents, including *Thrichomys laurentius*, *Rhipidomys sp.*, *Kerodon rupestris*, and *N. lasiurus*. Phylogenetic analysis placed the sequences within the *M. haemofelis* group but as distinct genotypes forming five clusters, with haplotype analysis indicating high genetic divergence. Based on these findings, two novel species were proposed: ‘*Candidatus* Mycoplasma haematorupestris’ (in *K. rupestris*) and ‘*Candidatus* Mycoplasma haematolaurentius’ (in *T. laurentius*). Although related to the ‘haemofelis group’, these genotypes showed marked genetic divergence, highlighting the remarkable diversity of hemoplasmas circulating among rodents.

Machado et al. (2024) screened Cricetidae rodents from the Atlantic Forest (Paraná and Rio de Janeiro) and detected hemoplasma DNA in multiple species, with an overall occurrence of 36.5%. Positive species included *Akodon* spp.*, Euryoryzomys russatus, N. lasiurus, Oligoryzomys* spp*., Oxymycterus* spp*., Sooretamys angouya*, and *haptomys nigrita*. Infection was significantly more frequent in males and in rodents of the genus *Oligoryzomys*, while no association was found with the presence of ectoparasites. Phylogenetic analysis of larger 16S rRNA fragments revealed two clades within the ‘haemofelis group’: one clade mainly containing *Akodon* spp. sequences, clustering with *Rattus rattus-*associated sequences from Brazil and Hungary; and another formed by *Oligoryzomys nigripes-*associated sequences, clustering with hemoplasmas from other wild rodents in Brazil, and closely related to ‘*Ca*. M. haemomuris subsp. musculi’.

Collectively, these studies demonstrate that hemoplasma infections are widespread and genetically diverse among rodents from Brazil, with both high-prevalence hosts (such as capybaras) and more restricted occurrences in sylvatic species. Notably, the recurrent detection of hemoplasma DNA in ticks and lice collected from infected rodents, together with the consistently high rates of ectoparasite infestation (Gonçalves et al., 2020; Valente et al., 2020; Vieira et al., 2021), supports a hypothesis that arthropods may play a role in the maintenance and transmission of these pathogens. However, more studies are necessary to confirm this assumption.

#### Didelphimorphia

The first report of hemoplasma detection in marsupials in Brazil was conducted by Massini et al. (2019), who investigated white-eared opossums (*Didelphis albiventris*) from an urban area in Paraná State (Atlantic Forest biome). The obtained sequences showed 98.97% similarity with *‘Candidatus* Mycoplasma haemodidelphis*’* and phylogenetic analysis positioned the sequences within the ‘suis group’, forming a sister clade to *‘Ca.* M. haemodidelphis’. In the following year, Gonçalves et al. (2020) reported similar results in white-eared opossums from the city of Campo Grande (Mato Grosso do Sul; Cerrado biome). Obtained sequences demonstrated 98.8% of similarity with *‘Ca. M. haemodidelphis’*, once again forming a sister clade in the phylogeny assessment. In both studies, infected animals were infested with *A. dubitatum*. However, the ticks were either not screened or tested negative for hemoplasma DNA.

Pontarolo et al. (2021) detected hemoplasmas in white-eared opossums sampled in the state of Santa Catarina (Atlantic Forest biome). Based on the phylogenetic positioning of extended fragments of the 16S and 23S rRNA genes and the geographic separation between *D. albiventris* and *D. virginiana*, the authors proposed a new *Candidatus* species: *‘Candidatus* Mycoplasma haemoalbiventris’. This genotype has been detected in *D. albiventris* populations from different Brazilian biomes, including the Atlantic Forest (Massini et al., 2019; Pontarolo et al., 2021; Oliveira et al., 2021) and Cerrado (Gonçalves et al., 2020).

Subsequently, Orozco et al. (2022) reported hemoplasma infection in *Didelphis aurita* from Minas Gerais. A prevalence 73.3% was observed, and the animals were infested with ticks of several species (*Amblyomma* spp., *Amblyomma ovale*, *A. dubitatum*, and *Ixodes loricatus*), although ectoparasites were not tested. Phylogenetic analysis based on partial 16S rRNA gene showed that *‘Ca.* M. haemodidelphis’ shares a common ancestor with a clade composed of hemoplasma sequences detected in *Didelphis* spp. in Brazil. This clade, in turn, was subdivided into two subclades: one containing *‘Ca.* M. haemoalbiventris*’* sequences and another composed of *D. aurita* sequences.

Later, Oliveira et al. (2023) reported the presence of hemoplasmas in *D. aurita* sampled in urban areas in Rio de Janeiro. The phylogenetic analysis of partial 16S rRNA gene supported two subclades within the *Didelphis* spp. hemoplasma clade in Brazil, with ‘*Ca*. M. haemodidelphis’ clustering closer to *D. aurita* sequences. In contrast, 23S rRNA analysis grouped *D. aurita* within the same clade as ‘*Ca*. M. haemoalbiventris’. The lack of 23S rRNA data for ‘*Ca*. M. haemodidelphis’ limited a more robust phylogenetic interpretation. Similarly, Machado et al. (2024) reported the detection of hemoplasmas in one free-ranging *D. aurita* in the state of Rio de Janeiro; however, a readable sequence from this amplicon was not obtained.

Braga et al. (2023) reported hemoplasma infection in *D. marsupialis* from the Amazon region (Maranhão). Based on partial 16S rRNA gene phylogeny, two major subclades were identified: one comprising *‘Ca.* M. haemoalbiventris*’* sequences and another containing sequences from *D. aurita* (Orozco et al., 2022; Oliveira et al., 2023) and *D. marsupialis*, which share a common ancestor with *‘Ca.* M. haemodidelphis*’*. The 23S rRNA-based phylogeny showed a similar pattern to that described by Oliveira et al. (2023), with lower differentiation between *D. albiventris* and *D. aurita/D. marsupialis* genotypes.

Torres-Santos et al. (2024) reported the occurrence of hemoplasmas in *D. albiventris* from the Caatinga biome. The phylogenetic inference based on 16S rRNA sequences grouped more closely with *‘Ca. M. haemodidelphis’*, forming a separate clade from *‘Ca.* M. haemoalbiventris*’*, with strong support. However, sequences obtained in previous studies from Minas Gerais (Orozco et al., 2022), Rio de Janeiro (Oliveira et al., 2023), and Maranhão (Braga et al., 2023) were not included, limiting further phylogenetic conclusions regarding this positioning.

Recently, Lignon et al. (2025) reported a high prevalence of hemoplasmas in road-killed *D. albiventris* in the Pampa biome (Rio Grande do Sul). However, phylogenetic assessment was performed based on 23S rRNA fragments only. As previously demonstrated by Oliveira et al. (2023) and Braga et al. (2023), comparisons between the 16S and 23S rRNA genes may reveal topological inconsistencies in phylogenetic trees for hemoplasmas obtained from *Didelphis* spp., possibly due to the higher sequence variability of the 23S rRNA gene and the limited availability of 23S rRNA sequences in public databases. On the other hand, a recent study conducted in the Amazon biome reported the amplification of hemoplasma 16S rRNA fragments from *D. marsupialis* samples, although no readable sequences were obtained. Alternatively, a hemoplasma 23S rRNA fragment was successfully amplified and sequenced, confirming the occurrence of hemotropic *Mycoplasma* spp (Chagas-de-Souza et al., 2025). This finding highlights the importance of this genetic target as an alternative marker for hemoplasma detection and phylogenetic reconstructions.

The prevalence of hemoplasmas in *Didelphis* spp. in Brazil varies widely, from 15.6% to 87.5% in studies using conventional PCR (Massini et al., 2019; Gonçalves et al., 2020; Oliveira et al., 2021; Orozco et al., 2022; Braga et al., 2023) and from 19% to 90% in those using qPCR (Torres-Santos et al., 2024; Lignon et al., 2025). Apparently, these variations are more strongly associated with environmental and anthropogenic factors than with methodological differences. In contrast, reports of hemoplasma infection in non-*Didelphis* marsupials are scarce. Samplings of *Thylamys macrurus, Gracilinanus agilis,* and *Monodelphis domestica* in the Pantanal (Sousa et al., 2017), *Monodelphis americana* in Rio de Janeiro, and *Philander quica* in Paraná State (Machado et al., 2024) found no evidence of hemoplasma infection. The only report of hemoplasma detection occurred in *M. domestica* from the Caatinga biome, with low prevalence (Torres-Santos et al., 2024). Moreover, the sequence obtained from *M. domestica* grouped closely with hemoplasmas detected in wild rodents (*T. laurentius*) from the same region, suggesting possible cross-species transmission.

Taken together, these studies support the occurrence of at least one genotype phylogenetically distinct from *‘Ca.* M. haemodidelphis’ in Brazilian marsupials. *‘Ca.* M. haemoalbiventris’ has been reported in *D. albiventris* from the southern and central-western regions of Brazil, including Atlantic Forest, Cerrado, and Pampa areas. A second genotype, more closely related to *‘Ca.* M. haemodidelphis’, may be circulating in *D. aurita* and *D. marsupialis*, although further studies, ideally including complete genome sequencing and broader geographic sampling, are needed to confirm this hypothesis.

Regarding transmission, although studies reported infestation of infected hosts by ticks (Gonçalves et al., 2020; Orozco et al., 2022) and fleas (Oliveira et al., 2021), no statistical associations or detection of hemoplasma DNA in ectoparasites from opossums have been documented. The clinical relevance of hemoplasma infection in opossums is unknown. In the study by Orozco et al. (2022), hemoplasma-positive animals showed significantly lower platelets counts, while total plasma protein concentration and alkaline phosphatase activity was significantly increased.

#### Superorder Xenarthra

##### Pilosa and Cingulata

The first report of hemoplasmas in Brazilian xenarthrans was provided by Oliveira et al. (2022), who screened different species from multiple biomes in different states (Amazon, Pantanal, Cerrado, Atlantic Forest, and Pampa from states of Mato Grosso do Sul, São Paulo, Pará, Rondônia, and Rio Grande do Sul) and found an overall prevalence of approximately 16%. Positive species included three-toed sloth (*Bradypus tridactylus* and *Bradypus* sp.), two-toed sloth (*Choloepus* sp.), lesser anteater (*Tamandua tetradactyla*), giant anteater (*Myrmecophaga tridactyla*), six-banded armadillo (*Euphractus sexcinctus*), nine-banded armadillo (*Dasypus novemcinctus*), and giant armadillo (*Priodontes maximus*); while southern naked-tailed armadillo (*Cabassous unicinctus*) and two-toed sloth (*Choloepus didactylus*) were negative. The study described three new genotypes, with two being proposed as candidate species: ‘*Candidatus* Mycoplasma haematotetradactyla’ obtained from *T. tetradactyla*, ‘*Candidatus* M. haematomaximus’ obtained from *P. maximus*, and *Mycoplasma* sp. obtained from *B. tridactylus*. In the phylogenetic analysis based on the 16S rRNA gene, all genotypes were positioned into the ‘suis group’. The clades of ‘*Ca*. M. haematotetradactyla’ and ‘*Ca*. M. hematomaximus’ were closely positioned, albeit separated by high bootstrap values. Interestingly, the sequence found in *B. tridactylus* was positioned in the same clade as *M. wenyonii* found in bovines.

Later, Sada et al. (2024) reported 52% of hemoplasmas in armadillos (*E. sexcinctus*, *P. maximus,* and *D. novemcinctus*) and anteaters (*M. tridactyla* and *T. tetradactyla*) collected in the states of Mato Grosso do Sul and São Paulo. Most of the positive animals were from Mato Grosso do Sul state, with one positive *M. tridactyla* from São Paulo state. The 16S rRNA-based phylogenetic analysis revealed that sequences obtained from *E. sexcinctus* and *P. maximus* from Mato Grosso do Sul clustered in the same clade of ‘*Ca*. M. haematomaximus', previously described in *P. maximus* from the same state (Oliveira et al., 2022). This report shows that the six-banded armadillo can be a host of ‘*Ca*. M. haematomaximus'.

Hemoplasma DNA was recently detected in silky anteaters (*Cyclopes* spp.) from northern and northeastern Brazil. A prevalence of 58.3% positivity was reported in *Cyclopes didactylus*, *Cyclopes thomasi*, and *Cyclopes rufus* (Rodrigues et al., 2025). These strictly arboreal xenarthrans from the order Pilosa remain poorly studied. Phylogenetic analysis based on partial 16S rRNA gene revealed that hemoplasmas from *C. didactylus* clustered with sequences previously obtained from *Coendou* sp. in Brazil. Another sequence grouped closely with hemoplasmas from *Alouatta* sp., albeit with low bootstrap support. Interestingly, both *Coendou* sp. and *Alouatta* sp. are arboreal species, suggesting that shared behavioral traits may facilitate transmission. Additionally, a third sequence clustered within the same clade as ‘Ca. M. haematobovis’ and a sequence obtained from *M. tridactyla* in Brazil. Similar to the findings of Oliveira et al. (2022), this suggests that cattle-like hemoplasmas may also circulate among xenarthrans.

Altogether, these findings demonstrate that at least two distinct hemoplasma genotypes circulate among wild xenarthrans from different biomes in Brazil: ‘*Ca.* M. haematomaximus’ has been detected in *P. maximus* and *E. sexcinctus* from the Pantanal and/or Cerrado biomes (the precise biome was not specified, but samplings were conducted in Mato Grosso do Sul), whereas ‘*Ca.* M. haematotetradactyla’ was found in *T. tetradactyla* from Amazon (Pará), Cerrado (Mato Grosso do Sul), and Pampa (Rio Grande do Sul) regions. Additionally, hemoplasmas related to other hosts were detected in *C. didactylus* from northern and northeastern regions (biomes not specified but among Amazon, Cerrado, and Atlantic Forest), suggesting possible cross-species transmission. However, further studies are required to confirm this hypothesis. Similarly, the genotypes detected in *B. tridactylus* and *C. didactylus*, which clustered near bovine-associated hemoplasmas (*M. wenyonii* and ‘*Ca. M. haematobovis*’, respectively), need further investigation to determine whether they represent novel genotypes or spillover events. No information on clinical or hematological changes in hemoplasma-infected xenarthrans is available.

### Aquatic mammals

Aquatic mammals are distinct from terrestrial mammals not only because they are adapted to live in marine environments, but also due to their unique evolutionary history. Phylogenetically, these animals belong to specific mammalian orders that diverged from other lineages millions of years ago (Supin et al., 2001). Currently, there are reports of hemoplasma detection in freshwater and marine mammals in Brazil.

Duarte-Benvenuto et al. (2022) detected hemoplasma DNA in wild river dolphins from the Amazon River Basin. Notably, positivity rates of 61-65.6% were observed for Amazon river dolphins (*Inia greoffrensis*) and Bolivian river dolphins (*Inia bolivensis*). Despite the additional sampling of captive river manatees (*Trichechus inunguis*), no molecular amplification was observed for these animals. Although phylogenetic analysis was not performed, the 16S rRNA network analysis revealed at least three distinct groups: one associated with Amazon river dolphins and others comprising highly diverse genotypes linked to Bolivian river dolphins.

Regarding marine mammals, another study demonstrated the occurrence of hemoplasmas in wild cetaceans and pinnipeds sampled alongside different coastal areas in Brazil (northeastern and southeastern regions) (Duarte-Benvenuto et al., 2023). For cetaceans, a positivity rate of 13.8% was obtained from species belonging to the Kogiidae, Delphinidae, and Pontoporidae families. Regarding pinnipeds, a positivity rate of 16.6% was observed for subantarctic fur seal (*Arctocephalus tropicalis*) and antarctic fur seal (*Arctocephalus gazelle*). The sequences from cetaceans showed 98.4-100% of identity with sequences from river dolphins from the Amazon River Basin, while sequences detected in subantarctic fur seals presented 99.3-100% identity with sequences obtained from Californian sea lion (*Zalophus californianus*) in the USA. Similar to the previous study, captive marine manatees (*Trichechus manatus*) were sampled, but amplification of hemoplasma DNA was not observed.

In southern Brazil (Rio Grande do Sul state), Battisti et al. (2024) investigated hemoplasmas in carcasses of pinnipeds, including South American fur seal (*Arctocephalus australis*), subantarctic fur seal, antarctic fur seal, and South America sea lion (*Otaria flavescens*). Hemoplasma DNA was detected in only 2.2% of South American fur seal samples. Phylogenetic analysis based on 16S rRNA gene positioned the obtained sequences within the 'suis group', forming a sister clade to '*Candidatus* Mycoplasma haemozalophi', previously identified in Californian sea lion in the USA and subantarctic fur seal from Brazil (Duarte-Benvenuto et al., 2023). Haplotype network analysis showed separation by a median vector, supporting the occurrence of a potentially distinct genotype in South American fur seal, possibly influenced by host geographic distribution.

Considering these reports, hemoplasmas occur both in river dolphins from the Amazon Basin and in cetaceans and pinnipeds from different coastal regions of Brazil. Among freshwater mammals, at least one hemoplasma genotype is associated with Amazon river dolphins, while at least two distinct genotypes are linked to Bolivian river dolphins. In marine mammals, hemoplasmas genetically similar to those found in river dolphins*,* and apparently positioned within the ‘suis group', have been detected in different cetacean species, including members of the family Delphinidae and the genera *Kogia* and *Pontoporia*. In fur seals, at least two distinct genotypes seem to occur along the Brazilian coast, associated with different host species. However, phylogenetic inferences are still needed to draw more robust conclusions.

Although adults showed significantly higher prevalence than calves, no significant differences were identified in hematological parameters between hemoplasma-positive and negative river dolphins (Duarte-Benvenuto et al., 2022). In contrast, age did not significantly affect hemoplasma detection in marine mammals (Duarte-Benvenuto et al., 2023). The pathogenicity of these infections remains to be elucidated.

### Flying mammals (order Chiroptera)

Bats are the second-largest order of mammals globally, comprising approximately 20% of mammalian biodiversity and playing essential ecological roles in pollination, seed dispersal, and regulating insect populations (Schipper et al., 2008; Kunz et al., 2011). Beyond their environmental importance, bats are also recognized as reservoirs for a wide range of pathogens, including hemotropic *Mycoplasma* spp. While the exact transmission dynamics of hemoplasmas in bats remain poorly understood, potential routes include ectoparasite vectors (Hornok et al., 2019) and social behaviors, such as biting and grooming, particularly in hematophagous species (Volokhov et al., 2017). In fact, a multilayer network analysis showed that hemoplasmas were restricted to the bat-bacteria layer, with no occurrence in ectoparasite-bacteria interactions. This finding suggests that transmission by hematophagous arthropods is not the main route of dissemination of these agents among bats. The detection of hemoplasmas DNA in ectoparasites was explained as vertebrate blood remnants in the digestive tract and might not represent evidence of active vectorial transmission (Alcantara et al., 2023).

Ikeda et al. (2017) screened species across four families (Vespertilionidae, Phyllostomidae, Molossidae, and Natalidae) in the states of Mato Grosso, Pará, Paraná, São Paulo, and Tocantins. Hemoplasmas were detected in 13.97%, mainly in individuals from Paraná, particularly in *Molossus molossus*, *Sturnira lilium*, and *Eptesicus* spp. Due to the low quality of some amplified products, only five hemoplasma 16S rRNA sequences were obtained (from Paraná and Pará), all from *M. molossus* specimens, and presenting low similarity rates (93-96%) with *M. coccoides*. Phylogenetically, these sequences clustered within the ‘haemofelis group’, and positioned in a clade closely related to *M*. *coccoides* and hemoplasma genotypes previously reported in rodents from Brazil, indicating potential evolutionary links between bat and rodent-associated hemoplasmas. This study also reports the first molecular detection of hemoplasmas in eight bat species (*Artibeus planirostris*, *Eptesicus* sp., *Eumops auripendulus*, *Glossophaga soricina*, *M. molossus*, *Molossus rufus*, *Myotis nigricans*, and *S. lilium*).

Expanding on these findings, Santos et al. (2020) investigated hematophagous (*Desmodus rotundus*, *Diphylla ecaudata*) and non-hematophagous (*Molossus* sp.) bats from Paraná State and detected hemoplasma DNA in a high proportion of samples (80%). Phylogenetic analyses revealed multiple genotypes, including sequences from vampire bats clustering with genotype I previously reported in *D. rotundus* from Peru and Belize (Volokhov et al., 2017). Other sequences from hematophagous bats clustered with genotype III previously described in *D. rotundus* from Peru and were closely related to hemoplasmas detected in coatis and capybaras from the same region (Vieira et al., 2009; Cubilla et al., 2017a). In addition, sequences obtained from *Molossus* sp. grouped with hemoplasmas previously reported in this host (Ikeda et al., 2017). Also, in another study from Paraná, one bat (*Artibeus lituratus*) was positive for hemoplasmas (Collere et al., 2022). The sequence clustered within the *M*. *haemofelis* group and showed high similarity to hemoplasmas detected in *Sturnira parvidens* and *Uroderma bilobatum* from Belize.

In central-western Brazil, a molecular survey reported hemoplasmas in 43% of non-hematophagous bats (*A. planirostris*, *Platyrrhinus discolor*, *A. lituratus*, *Platyrrhinus lineatus*, *Carollia perspicillata*, *Eptesicus furinalis*, *M. molossus*, *Chiroderma villosum*, and *G. soricina*) (Ikeda et al., 2022). Additionally, ectoparasites were collected from bats, and 1.6% of associated bat flies (*Megistopoda aranea*) were positive. Phylogenetic analyses placed the 16S rRNA sequences within ‘haemofelis group’, clustering with previously described bat-associated hemoplasmas in Brazil (Ikeda et al., 2017) and other parts of the world, indicating high genetic diversity of hemoplasmas among bats. In contrast, the 23S rRNA sequences formed a sister clade to ‘*Ca*. M. haematohydrochaerus’ and ‘*Ca*. M. haemosphiggurus'. Although bipartite network analysis indicated a slight tendency for phylogenetically related bat species to share similar hemoplasma genotypes, the network structure was too sparse to confirm any apparent species-level clustering.

De Mello et al. (2023) surveyed vampire bats (*Desmodus rotundus*, *Diphylla ecaudata*, and *Diaemus youngi*) across all five regions of Brazil and found a lower prevalence (6%) compared to previous reports, with most positives in *D. rotundus*, one in *D. ecaudata*, and none in *D. youngi*. Phylogenetic analysis positioned the two obtained sequences (one from São Paulo and the other from Tocantins) in different clades within the ‘haemofelis group’, clustering together with sequences of vampire and non-hematophagous bats from other parts of the world, which reinforces the circulation of phylogenetically conserved genotypes across different regions. Authors attribute the low prevalence to the type of sample used (liver), since increased prevalence rates were obtained with blood or spleen samples (Volokhov et al., 2017; Santos et al., 2020; Ikeda et al., 2022).

De Mello et al. (2024) investigated the presence of hemoplasma DNA in *D. rotundus* and *D. youngii* from northern Brazil, identifying 10% positive *D. rotundus*, and no positive samples from *D. youngii*. Despite the use of spleen samples, the observed prevalence was considered lower than that observed in southern Brazil. Following a similar pattern from previous studies, the obtained sequences presented >98% of similarity with sequences obtained from *D. rotundus* in Belize. Regarding phylogenetic assessment, sequences were positioned within the ‘haemofelis group’, and genotype network analyses consistently revealed high diversity, with multiple distinct genotypes observed across sites.

Recently, Silva et al. (2025) reported a prevalence of 40% of hemoplasmas in non-hematophagous bats from the Brazilian Amazon (Acre State). Positive species included *Anoura caudifer*, *A. lituratus*, *A. planirostis*, *Carollia beikeith c.f.*, *C. perspicillata*, *Carollia brevicauda*, *Dermanura cinereus*, *G. soricina*, *Lophostoma silviculum*, *Phyllostomus discolor*, *Phyllostomus elongatus*, *Phyllostomus hastatus*, *Rhinophylla fischerae*, *Sturnira tildae*, and *Platyrhinus infuscus*. The hemoplasma genotypes from Acre clustered closely with previously reported lineages from both hematophagous and non-hematophagous bats (Oliveira et al., 2017; Ikeda et al., 2022; De Mello et al., 2023). Notably, phylogenetic clustering did not follow feeding habits, indicating that dietary habits may not significantly influence hemoplasma lineage separation among bats.

Together, the findings across different studies conducted in Brazil highlight the remarkable genetic diversity and widespread occurrence of bat-associated hemoplasmas. The genotypic analyses, particularly those based on the 16S rRNA gene, revealed a high number of distinct genotypes (even within the same locality or host species), indicating dynamic evolutionary patterns and possible host-specific associations. This pattern of diversity has been consistently observed in hematophagous bats such as *D. rotundus* (De Mello et al., 2023, 2024) and in non-hematophagous species across different biomes (Ikeda et al., 2017; Silva et al., 2025).

### Exotic species

Hemoplasma detection was reported in exotic mammal species maintained in zoos (André et al., 2011; Castillo et al., 2024), laboratory facilities (Mongruel et al., 2022a), and free-ranging animals (Dias et al., 2019; Santana et al., 2022) in Brazil.

Wild boars (*Sus scrofa*) are considered one of the worst invasive species in the world. In Brazil, it represents a threat to biodiversity and is reported to occur in at least 15 states (Kaizer et al., 2014; Brasil, 2025). Dias et al. (2019) reported the occurrence of *M. suis* in wild boars hunted in the state of São Paulo. The authors observed a 50% prevalence of positive animals. However, the bacterial load was low, indicating low levels of bacteremia and suggesting that these boars may act as chronic carriers of the pathogen. Fernandes et al. (2022) demonstrated the occurrence of hemoplasmas in wild boar populations sampled in the South (Atlantic Forest) and Central-West (Cerrado) regions of Brazil, with a 58.5% prevalence of positivity and no statistical difference between the two biomes. Analysis of the obtained sequences revealed the presence of *M. suis*, *M. parvum*, and two sequences with <93% identity to these species, suggesting the occurrence of a potential novel hemoplasma species in wild boars. Although hunting dogs also tested positive in the same study, the sequences obtained from them were distinct, with high similarity to *M. haemocanis*, suggesting the absence of cross-transmission between wild boars and dogs.

Santana et al. (2022) described a higher prevalence of hemoplasmas (88%) in wild boars sampled in São Paulo state. Furthermore, ticks (*A. sculptum* and *A. ovale*) collected from these wild boars also tested positive, although with lower prevalence (8.7%). Considering these values and the lack of evidence of *M. suis* transmission by arthropod vectors, the DNA detection in ticks may be associated with residual presence after a blood meal rather than vectorial competence. Phylogenetic inference of the 23S rRNA gene revealed genotypes related to *M. suis* and *M. parvum*, with the detection of two distinct genotypes in the same animal. The studies included herein reported hemoplasma prevalence rates ranging from 50% to 88% in wild boars sampled in the Atlantic Forest and Cerrado biomes in Brazil. Sequences with high similarity to *M. suis* and *M. parvum* were identified, along with potentially novel genotypes.

Rhesus monkeys (*Macaca mulatta*) maintained in a laboratory colony were reported as hosts of hemoplasmas in Rio de Janeiro, Brazil. Mongruel et al. (2022a) described notable frequencies of infection (62.5%-66.7%) in these animals, with no previous history of ectoparasites, which reinforces the hypothesis of vertical or through direct contact transmission (Millán et al., 2021). In the phylogenetic analyses, sequences were grouped in the same clade of ‘*Ca*. M. haematomacacae’ from *Macaca* species from Japan and the USA, within the ‘haemofelis group’, and forming a distinct clade from the hemoplasmas identified in *Sapajus* spp. in Brazil (Bonato et al., 2015; Ramalho et al., 2017). This finding reinforces the difference between a potential *Sapajus*-related hemoplasma species and ‘*Ca*. M. haematomacacae’, albeit phylogenetically related. Although the infection was not associated with anemia or relevant hematological alterations in the evaluated animals, this finding highlights the potential of these microorganisms to act as confounding variables in experimental studies.

Regarding ruminants**,**Santos et al. (2017) reported the absence of hemoplasma detection in Babery sheep (*Ammotragus lervia*) kept in captivity in a zoo in southern Brazil. Recently, a case of hemoplasma infection was recorded in an exotic deer species (*Dama dama*) housed at a zoo in Minas Gerais state, southeastern Brazil. Although the report was based solely on DNA amplification, without sequencing, it supports the circulation of hemotropic *Mycoplasma* spp. in exotic cervids maintained in captivity (Castillo et al., 2024).

In non-native wild felids, hemoplasma infections were reported in lions (*Panthera leo*) (Guimarães et al., 2007b; Willi et al., 2007a; Carneiro et al., 2020) kept in zoos from southern and central-west regions of Brazil. All studies report a lack of clinical signals, emphasizing that infections may often go unnoticed in zoo settings. Infections have also been documented in non-native canids in Brazil, as two/three gray wolves (*Canis lupus*) housed at a zoo in São Paulo state were reported positive for hemoplasmas genetically similar to ‘*Ca*. M. haematominutum’ (André et al., 2011).

Conrado et al. (2015) reported the first hemoplasma infection in rats from Brazil. Blood samples from *Rattus norvegicus* were collected from two locations in Curitiba, southern Brazil: a public park and the city zoo, along with laboratory-raised rats used for carnivorous feeding. The overall prevalence was 63.5%, with no statistical difference between the groups. Most obtained sequences showed high percentage identities (98-100%) with *M. haemomuris*. Phylogenetic analysis positioned the sequences in distinct clades within the ‘haemofelis group’: sequences were grouped with *Mycoplasma* sp. of Eurasian harvest mouse (*Micromys minutus*) from Hungary, forming a sister clade to hemoplasmas from capybaras in Brazil, while other sequences clustered with *M. haemomuris*. The results indicate that *M. haemomuris* may be endemic in rodents in the region, including laboratory rats, and suggest the circulation of a potential novel genotype or species. No significant hematological changes were observed in positive animals.

Similarly, synanthropic rodents, namely *R. rattus* and *Mus musculus,* were positive for hemotropic *Mycoplasma* spp. in a study analyzing samples from native and non-native species of wild rodents from Brazil (Gonçalves et al., 2015). Interestingly, sequences obtained from *R. rattus* did not group with those of native Brazilian rodents but instead clustered with strains previously described in Asia and Europe, supporting the hypothesis that these hemoplasmas were introduced during historical colonization events. Among the synanthropic rodent sequences analyzed (*R. rattus*), some showed 99% identity to *‘Ca.* M. haemomuris subsp. ratti’ was previously detected in *R. rattus* from Japan, while others clustered with *Mycoplasma* sp. strains from *R. norvegicus* in Japan and Hungary. Later, Gonçalves et al. (2020) reinforced these findings by detecting hemoplasma DNA in *R. rattus* (but none of the *M. musculus*) sampled in central-western Brazil. Hemoplasma DNA was also found in *Amblyomma* sp. larvae and *Polyplax spinulosa* lice collected from *R. rattus*. Phylogenetic analysis confirmed close identity (99.5–100%) with murine hemoplasmas previously reported in Brazil, Japan, and Hungary, further reinforcing the likely non-native origin of these lineages (Gonçalves et al., 2015).

A recent study by Torres-Santos et al. (2024) reported two out of nine *R. rattus* positive for hemoplasmas. The phylogenetic analyses based on the 16S and 23S rRNA gene fragments revealed high identity (99.65–100%) with hemotropic *Mycoplasma* spp. and 99.89% identity with *‘Ca.* M. haemomuris subsp. ratti’ previously detected in *R. rattus* (Gonçalves et al., 2015, 2020). Similarly, Machado et al. (2024) reported the occurrence of hemoplasmas in two *R. rattus* from Paraná State. Although hemoplasma sequences from these synanthropic rodents were not obtained, phylogenetic analysis was built on sequences obtained from native wild rodent species (*Akdon* spp. and *Oligoryzomys nigripes*), which were positioned in clades closely related to hemoplasma sequences obtained from *R. rattus* and *R. novergicus* from Brazil and other countries. In contrast, haplotype network analysis revealed haplotypes exclusive to wild and synanthropic rodents, separated by intermediate mutations. These results suggest that hemoplasmas from wild and synanthropic rodents are genetically related but represent distinct lineages.

Together, these findings support the hypothesis that hemoplasmas detected in *R. rattus* represent non-native lineages likely introduced historically, in contrast to those circulating in native rodents, such as capybaras. This separation suggests potential host specificity and highlights the distinct evolutionary trajectories of hemoplasmas in synanthropic versus wild rodent populations in Brazil.

### Good practices in taxonomic proposals

The term *Candidatus* was implemented by the International Code of Nomenclature of Bacteria to designate new and incompletely described taxa, allowing a provisional name to organisms that have not yet been fully characterized or remain uncultivable. Although sequences of different macromolecules are allowed for the designation of *Candidatus* species, 16S rRNA sequences with at least 1,000 bp are considered mandatory (Murray & Stackebrandt, 1995). However, in practice, obtaining such long sequences can be particularly difficult when working with wildlife biological samples. Several factors, such as the amount of material available, sample transport and storage conditions, and financial or permit constraints, may prevent sampling, acquisition of DNA with satisfactory quality, or amplification efficiency.

Until today, it is established that novel genotypes of hemoplasmas continue to be described under *Candidatus* status until pure cultures and complete phenotypic characterizations are obtained, while already validated species should keep their formal names (Neimark et al., 2002). Although *Candidatus* names are considered provisional due to the impossibility of *in vitro* cultivation, proposals to accept genetic sequences as type material have been made (Whitman et al., 2019). Furthermore, even though *Candidatus* names do not have official status yet, it is recommended that they be formulated according to the rules of the INCP (Oren et al., 2020; Oren, 2021). However, these names frequently lack formal standing in prokaryotic nomenclature. In 2017, approximately 400 *Candidatus* names were evaluated and 120 were identified as inconsistent according to the International Code of Nomenclature of Prokaryotes (ICNP). Corrected forms were proposed in accordance with the grammatical and etymological rules. Although these corrections are not formally validated, they represent practical guidance for consistent and standardized use of *Candidatus* names (Oren, 2017). For practical purposes, the corrected forms may be adopted when referring to these organisms, and researchers should ensure that their consistent use prevents the correct names from becoming ambiguous once widely adopted.

Considering that most hemoplasma genotypes retrieved from wildlife are not yet fully characterized, it is recommended that authors use universal primers rather than species-specific primers to avoid underestimation due to protocol specificity limitations (Millán et al., 2021). All bacteria carry at least one copy of the 16S rRNA gene, whose conserved and hypervariable regions allow both broad bacterial detection and species-level identification. In this sense, although 16S rRNA is the most accepted initial marker to propose new bacterial species (with <97% similarity as a signal for a new taxon), it should be used together with other genetic and phenotypic data for a robust description (Drancourt & Raoult, 2005). Moreover, considering the evidence of highly variable genomes within the *Mycoplasma* genus (Citti et al., 2020), the use of characterization genes is useful for investigations of the genetic fingerprints of hemoplasmas. For these reasons, we strongly recommend that authors prioritize obtaining multilocus sequence data when proposing hemoplasma *Candidatus* species or genotypes. Additional genetic markers such as 23S rRNA (Mongruel et al., 2020), and *dnaK* (Descloux et al., 2021) may be used. According to Volokhov et al. (2023), the same genotype must be identified in at least two samples from the same host species (based on 16S rRNA and 23S rRNA gene sequences) to establish a novel *Candidatus* species.

Few studies have included morphologic information in their reports (Cubilla et al., 2017a, 2017b; Fagundes-Moreira et al., 2023). In this context, we also encourage authors to include as much phenotypic, structural, and ecological information as possible, considering that even for *Candidatus* taxa, molecular data needs to be supplemented with descriptive characteristics (Murray & Stackebrandt, 1995).

Regarding protocol selection, our experience indicates that some 16S rRNA primers are capable of cross-amplifying DNA from mucosal mycoplasmas or other genera. This is also reported by other studies (Millán et al., 2021). Nevertheless, this is not surprising considering the conservative nature of the 16S rRNA gene. In fact, hemoplasma-designated primers can produce enhanced prevalence through non-specific amplification (Moore et al., 2024). Sampling efforts are also significant for investigating the diversity of hemoplasmas in wildlife in depth. As exemplified by Mongruel et al. (2022b) and Baggio-Souza et al. (2025), wild populations of the same species living in the same area can be infected by genetically distinct hemoplasmas.

Finally, considering the increasing number of genotypes and *Candidatus* species of hemoplasmas reported in wildlife from Brazil, researchers should be aware of including a broad range of available sequences in their analysis to enhance the accuracy of phylogenetic positionings. The selection of sequences has a direct impact on the resulting topology, especially in analyses based on conserved genes such as the 16S rRNA. When key species or genotypes are not included, it can lead to misinterpretations of the evolutionary relationships among the analyzed taxa. Efforts to include an expanded number of taxa tend to improve the accuracy of the phylogenetic tree by reducing systematic bias and increasing resolution of clusters (Heath et al., 2008; Wiens & Tiu, 2012).

### Potential limitations of the present review

The present scoping review has potential limitations that need to be acknowledged. Considering that the main objective was to map the available evidence of hemoplasmas in Brazilian wildlife, no qualitative assessment or risk of bias analysis was performed. Our review included only reports published in scientific journals, and the included studies showed marked heterogeneity regarding host species, sampling design, molecular targets, and diagnostic protocols, which limits direct comparisons across reports. In addition, publication bias and uneven geographic distribution of research efforts may influence the patterns of occurrence.

## Conclusion

The present review assembled updated information on the occurrence and diversity of hemoplasmas in Brazilian wildlife. Based on the reviewed data, some main points can be highlighted:

As suggested by previous studies, hemoplasmas seem to co-evolve with their hosts. This pattern is evident in different mammalian species (e.g., *N. nasua*, *T. terrestris*, *D. albiventris*, *Sapajus* spp., *H. hydrochaeris*, and some species of aquatic mammals), which have been reported to harbor the same or closely related species/genotypes across different studies and biomes. This co-evolving hypothesis is strengthened by the phylogenetic positioning of hemoplasmas detected in exotic species, such as rhesus monkeys and synanthropic rodents, whose sequences cluster more closely with those from other countries than with sequences from native Brazilian primates or rodents. On the other hand, evidence of cross-transmission among marsupials and rodents, as well as between *C. thous* and *N. nasua*, suggests that not all wildlife hemoplasmas remain strictly species-specific. Additionally, the detection of genotypes related to cattle-associated species in xenarthrans raises concerns about potential spillover events and their implications for wildlife health, particularly in the Pantanal biome.

Despite the continental dimension of Brazil, most reports are concentrated in four states: 11 reports in São Paulo (Atlantic Forest), Paraná (Atlantic Forest), 10 reports in Mato Grosso do Sul (Pantanal/Cerrado), and seven reports in Rio Grande do Sul (Atlantic Forest/Pampa). However, the low number of reports in the other areas may reflect limited sampling effort and concentration of research groups rather than a true low prevalence.

Hemoplasma genotypes from rodents and bats stand out as notably genetically diverse. This may partly reflect higher sampling effort in these hosts, which allows the detection of a broader range of genotypes. Still, many lineages of hemoplasmas in wild animals from Brazil remain poorly characterized. Future studies should prioritize obtaining near-complete (or at least extended) fragments from the 16S rRNA gene, characterize genotypes by amplifying additional molecular markers (e.g., 23S rRNA gene), and include a broad range of taxa and previously reported genotypes from Brazilian wildlife in their phylogenetic inferences to generate more robust topologies. At the same time, metagenomic-assisted genomics may emerge as a promising approach to overcome the limitations of the current molecular techniques, especially considering the inability to cultivate hemoplasmas *in vitro*. These approaches are key to clarifying the evolutionary relationships and taxonomic placement of the reported lineages, helping to unravel the genetic puzzle of hemoplasmas from Brazil and worldwide.

## Data Availability

All available data is presented in the text.

## Supplementary Material

Supplementary material accompanies this paper.

Supplementary File 1 Reports of hemotropic Mycoplasma spp. in exotic animal species in Brazil. Figure was created using QGIS 3.28 software (http://qgis.org) and Canva Pro (http://canva.com).

Supplementary File 2 Map showing the geographical distribution of Brazilian biomes across States. Figure was created using QGIS 3.28 software (http://qgis.org) and Canva Pro (http://canva.com).

Supplementary File 3 Studies conducted in Brazil on the detection and occurrence of hemoplasmas in wildlife

This material is available as part of the online article from https://doi.org/10.1590/S1984-29612026007
