# Supplementary material for: Unveiling the diversity of hemoplasmas (hemotropic Mycoplasma spp.) in Brazilian wildlife: two decades of molecular discoveries – a scoping review
Source: Rev Bras Parasitol Vet. 2026 May 11;35(1):e017725. doi: 10.1590/S1984-29612026007 (PMC13193329; doi:10.1590/S1984-29612026007)
Supplement: Supplementary File 1 [file rbpv-35-1-e017725-suppl01.pdf]

Table 1. Studies conducted in Brazil on the detection and occurrence of hemoplasmas in wildlife.

Order	Family	Host species	Technique (Target gene)	Size of the obtained sequences or amplified fragment (bp)	Type of sample analyzed and positive in screenin g	No. of animals positive in screening / No. of animals analyzed (%)	*Mycoplasma* species and/or phylogenetic placement.	Sample origin – Brazilian state (Captivit y-C or Free-ranging-FR)	Clinica l signs and/or lesions	Reference
					**Mammals**					
Artiodactyla	Cervidae	*Blastocerus dichotomus* (Marsh deer)	cPCR (16S rRNA, 23S rRNA)	~1300 - 16S rRNA, 23S rRNA	Blood	4/6 (66.7%)	*M. ovis* phylogenetically related to *M. ovis-like*	PR (C)	No	Grazziotin et al. (2011a)
			cPCR (16S rRNA, 23S rRNA)	~1300 - 16S rRNA, 23S rRNA	Blood	46/64 (66.7%)	*M. ovis* phylogenetically related to *M. ovis-like, 'Ca.* M. erythrocerva'	SP (FR)	No	Grazziotin et al. (2011b)
		Mazama nana (Pygmy brocket)	cPCR (16S rRNA, 23S rRNA)	1300 - 16S rRNA, 23S rRNA	Blood	21/31 (95.5%)	*M. ovis* phylogenetically related to *M. ovis-like*	PR (C)	No	Grazziotin et al. (2011a)
		Mazama americana (red brocket deer)	cPCR (16S rRNA, 23S rRNA)	1300 - 16S rRNA, 23S rRNA	Blood	2/3 (66.7%)	*M. ovis* phylogenetically related to *M. ovis-like*	PR (C)	No	Grazziotin et al. (2011a)
			cPCR (16S rRNA)	~1300	Blood	1/3 (33.3%)	*M. ovis*	PR (FR)	No	André et al. (2020)
		Ozotoceros bezoarticus (Pampas deer)	cPCR (16S rRNA, 23S rRNA)	~1300 - 16S rRNA, 23S rRNA	Blood	14/39 (35.8%)	*M. ovis* phylogenetically related to *M. ovis-like, 'Ca.* M. erythrocerva'	MS (FR – 18 animals) GO (FR – 21 animals)	No	Grazziotin et al. (2011b)

			cPCR (16S rRNA)	~1300	Blood	5/11 (45.4%)	*M. ovis*	MS (FR)	No	André et al. (2020)
		Mozama gouazoubira (gray brocket deer)	cPCR (16S rRNA)	~1300	Blood	11/27 (40.7%)	*M. ovis*	MS/GO /SP (FR)	No	André et al. (2020)
		Mozama bororo	cPCR (16S rRNA)	~1300	Blood	1/4 (25%)	*M. ovis*	SP (FR)	No	André et al. (2020)
	Tayassuidae	*Pecari tacaju* (collared peccary)	cPCR (16S rRNA)	-	Blood	12/24 (50%)	No sequence	MG (C)	No	Silveira et al. (2024)
Carnivora	Canidae	*Cerdocyon thous* (crab-eating fox)	cPCR (16S rRNA, RNaseP)	~1000 – 16S rRNA	Blood	3/78 (3.8%)	*Mycoplasma spp. - 100% identity M. haemocanis*	DF (C)	No	Sousa et al. (2017)
			cPCR (16S rRNA)	-	Blood	4/5 (80%)	*C*Mhm, *M. haemocanis*	DF (C)	Yes (1 animal - coinfected)	Carneiro et al. (2020)
			cPCR (16S rDNA, 23S rRNA)	~800 – 23S rRNA	Blood	5/7 (71.4%)	*Mycoplasma sp.*	RS (FR – carcass of road-killed)	No	Lignon et al. (2025)
		Chrysocyon brachyurus (Maned wolf)	cPCR (16S rRNA)	-	Blood	6/6 (100%)	*Mycoplasma spp.*	DF (C)	Yes (1 animal - coinfected)	Carneiro et al. (2020)
			cPCR (16S rRNA)	-	Blood	1/5 (20%)	No sequence	MG (C)	No	Castillo et al. (2024)

	Lycalopex gymnocercus (Pampas fox)	cPCR (16S rDNA, 23S rRNA)	~800 – 23S rRNA	Blood	1/1 (100%)	*Mycoplasma sp.*	RS (FR – carcass of road-killed)	No	Lignon et al. (2025)
	Lycalopex vetulus (Hoary fox)	cPCR (16S rRNA)	-	Blood	4/5 (80%)	*M. haemocanis*	DF (C)	No	Carneiro et al. (2020)
	*Canis lupus** (European wolf)	cPC (16S rRNA)	~600	Blood	2/3 (66.6%)	CMhm (98% identity)	SP/MS/DF (C)	No	André et al. (2011)
	Speothos venaticus (Bush dogs)	cPC (16S rRNA)	~600	Blood	2/27 (7.4%)	'*Ca.* M. haematoparvum' (99% identity)	SP/MS/DF (C)	No	André et al. (2011)
		cPCR (16S rRNA)	-	Blood	1/2 (50%)	'*Ca.* M. haematoparvum'	DF (C)	No	Carneiro et al. (2020)
Felidae	*Leopardus pardalis* (ocelot)	qPCR (16S rRNA)<br>cPCR (16S rRNA, RNaseP)	~1400 – 16S rRNA	Blood	4/7 (57.1%)	CMh, CMhm, CMt	SP (C)	No	Willi et al. (2007)
		cPCR (16S rRNA)	~600	Blood	10/43 (23.2%)	CMhm (99% identity)	SP/MS/DF (C)	No	André et al. (2011)
		cPCR (16S rRNA, RNaseP)	~1000 - 16S rRNA	Blood	1/7 (14.2%)	*Mycoplasma spp.* - 99% identity CMhm	MS (C)	No	Sousa et al. (2017)
		cPCR (16S rRNA)	-	Blood	2/2 (100%)	'*Ca.* M. haematoparvum'	DF (C)	No	Carneiro et al. (2020)
	Leopardus tigrinus (oncilla)	qPCR (16S rRNA), cPCR (16S rRNA, RNase P)	~1400 – 16S rRNA	Blood	3/33 (9.09%)	CMhm	SP (C)	No	Willi et al. (2007)

	cPC (16S rRNA)	~600	Blood	4/39 (10.2%)	CMhm, Mhf (99% identity)	SP/MS/DF (C)	No	André et al. (2011)
Panthera leo (lion)*	qPCR (16S rRNA), cPCR (16S rRNA, RNase P)	-	Blood	1/4 (25%)	CMhm	SP (C)	No	Willi et al. (2007)
	cPCR (16S rRNA)	-	Blood	1/1 (100%)	No sequence	PR (C)	Yes	Guimarães et al., 2007
	cPCR (16S rRNA)	-	Blood	1/2 (50%)	No sequence	DF (C)	No	Carneiro et al. (2020)
Panthera onca (jaguar)	cPC (16S rRNA)	~600	Blood	4/14 (28.5%)	CMhm (99% identity)	SP/MS/DF (C)	No	André et al. (2011)
	cPCR (16S rRNA)	1.300 - 16S rRNA	Blood	1/1 (100%)	CMhm	PR (C)	No	Ribeiro et al. (2017)
	cPCR/nPCR (16S rRNA)	~250	Blood	22/30 (73.3%)	Mhf, CMhm, CMt	GO/MS/TO/AM (FR)	No	Furtado et al. (2018)
	cPCR (16S rRNA)	-	Blood	2/11 (18.1%)	CMhm, '*Ca.* M. haematoparvum'	DF (C)	No	Carneiro et al. (2020)
Puma concolor (puma)	qPCR (16S rRNA), cPCR (16S rRNA, RNase P)	-	Blood	1/2 (50%)	CMhm	SP (C)	No	Willi et al. (2007)
	cPCR (16S rRNA)	~600	Blood	3/18 (16.6%)	CMhm (99% identity)	SP/MS/DF (C)	No	André et al. (2011)

	cPCR (16S rRNA)	1.300	Blood	3/3 (100%)	CMhm	PR (C)	No	Ribeiro et al. (2017)
	cPCR (16S rRNA)	-	Blood	4/6 (66.6%)	*Mycoplasma sp.*	DF (C)	No	Carneiro et al. (2020)
Leopardus geoffroyi (Geoffroy cat)	qPCR (16S rRNA), cPCR (16S rRNA, RNase P)		Blood	1/7 (14.2%)	CMhm	SP (C)	No	Willi et al. (2007)
	cPCR (16S rRNA)	~600	Blood	30/60 (50%)	Mhf, CMt, CMhm (99.85–100% identity)	RS (FR)	No	Souza et al. (2025)
	cPCR (16S rDNA, 23S rRNA)	~800 – 23S rRNA	Blood	1/1 (100%)	CMhm	RS (FR – carcass of road-killed)	No	Lignon et al. (2025)
Leopardus wiedii (Margay)	qPCR (16S rRNA), cPCR (16S rRNA, RNase P)	~1400 – 16S rRNA	Blood	1/9 (11.1%)	Mhf, CMhm	SP (C)	No	Willi et al. (2007)
	cPCR (16S rRNA)	-	Blood	1/2 (50%)	*Mycoplasma sp.*	DF (C)	No	Carneiro et al. (2020)
	cPCR (16S rRNA)	~600	Blood	9/19 (47.4%)	Mhf, CMt, CMhm (99.85–100% identity)	RS (FR)	No	Souza et al. (2025)
Puma yagouaroundi (Jaguarondi*)*	cPC (16S rRNA)	~600	Blood	2/25 (8%)	CMhm (99% identity)	SP/MS/DF (C)	No	André et al. (2011)
	cPCR (16S rRNA)	-	Blood	2/3 (66.6%)	*Mycoplasma sp.*	DF (C)	No	Carneiro et al. (2020)
Leopardus pajerus (Pampa cat)	cPCR (16S rRNA)	-	Blood	3/3 (100%)	*M. haemocanis*, '*Ca.* M. haematoparvum'	DF (C)	No	Carneiro et al. (2020)

Mustelidae	L*ontra longicaudis* (neotropical otter)	cPCR (16S rRNA, 23S rRNA)	620 - 16S rRNA<br>800 - 23S rRNA	Blood, spleen and brain	11/14 (78.5%)	Novel - *'Ca.* M. haematocontesinii' (*M. felis* group) and '*Ca.* M. haematolongicaudis' (*M. suis* group)	MT/MS/RS/SC (FR)	No	Baggio-Souza et al. (2025)
	Galictis cuja (lesser grison)	cPCR (16S rRNA, 23S rRNA)	620 - 16S rRNA<br>800 - 23S rRNA	Blood, clot, spleen and liver	6/8 (75%)	*Novel* - *'Ca.* M. *haematohagiwarae'* (*M. suis* group)	MT/MS/RS/SC (FR)	No	Baggio-Souza et al. (2025)
Procyonidae	*Nasua nasua* (coati)	cPCR (16S rRNA, RNaseP)	~1000 - 16S rRNA	Blood	24/31 (74.4%)	*Mycoplasma spp.*<br>91% identity *M. haemofelis* - RNaseP	MS (FR)	No	Sousa et al. (2017)
		cPCR (16S rRNA) and Blood smears	~1300	Blood	2/ 2 (100%)	*Mycoplasma sp.* - >99% *M. haemofelis*	PR (C)	No	Cubilla et al. (2017b)
		cPCR (16S rRNA, 23S rRNA)	~800	Blood	8/18 (44.4%)	Novel - '*Ca.* M. haematonasua'	PR (FR)	No	Collere et al. (2021)
		qPCR (16S rRNA) cPCR (16S rRNA)	~646-1124 - cPCR 16S rRNA	Blood	86/97 (88.6%)<br>Genotype myc1 and myc2 (71% and 17%)	Myc1 - *'Ca.* M. haematonasua'<br>Myc2 - raccoon (P. lotor)-associated and M. haemofelis	MS (C)	No	Perles et al. (2023a)
		nPCR (16S rRNA)	~892 to 941	Blood	42/49 (85.7%)	99.8–100% identity '*Ca*. Mycoplasma haematonasua'	PR (C)	No	Perles et al. (2023b)

		Procyon cancrivorus (crab-eating Raccoon)	cPCR (16S rRNA)	600	Blood and spleen	6/9 (66.7%)	Genotype related to '*Ca*. Mycoplasma haemominutum'	RS/SC/PR (FR - 6 road-killed animals)	No	Fagundes-Moreira et al. (2023)
			cPCR (16S rDNA, 23S rRNA)	800 - 23S rRNA	Blood	1/1 (100%)	*Mycoplasma* sp.	RS (FR – carcass of road-killed)	No	Lignon et al. (2025)
	Otariidae	*Arctocephalus australis*	cPCR (16S rRNA, 23S rRNA)	~600 - 16S rRNA	Spleen	3/135 (2.2%)	*Mycoplasma* sp. - phylogenetically related to '*Ca*. M. haemozalophi' (*M. suis* group)	RS (FR)	No	Battisti et al. (2024)
Perissodactyla	Tapiridae	*Tapirus terrestris* (*lowland tapir*)	cPCR (16S rRNA, 23S rRNA, RNAse P, and dnaK)	1107 - 16S rRNA<br>~800 - 23S rRNA	Blood	36/99 (36.4%)	'Candidatus Mycoplasma haematoterrestris' - *M. suis* group<br>'Candidatus Mycoplasma haematotapirus' - *M. felis* group	MS (FR - 8 road-killed animals)	No	Mongruel et al. (2022b)
Didelphimorphia	Didelphidae	*Didelphis albiventris* (white-eared opossum)	cPCR (16S rRNA)	~800	Blood	7/8 (87.5%)	*Mycoplasma* spp. - 98.97% similarity '*Ca*. M. haemodidelphis'	PR (C)	No	Massini et al. (2019)
			cPCR (16S rRNA)	1200	Blood	14/43 (32.5%)	*Mycoplasma* spp. - 98.8% similarity '*Ca*. M. haemodidelphis'	MS (FR)	No	Gonçalves et al. (2020)
			cPCR (16S rRNA, 23S rRNA)	800 - 16S rRNA and 23S rRNA	Blood	20/50 (40%)	98.88% identity '*Ca*. M. haemodidelphis' (16S rRNA)	SC (FR - 17 road-killed animals)	No	Pontarolo et al. (2021)

					Novel - '*Ca*. M. haemoalbiventris'			
	cPCR (16S RRNA), cPCR (23S rRNA)	900 – 16S rRNA<br>800 – 23S rRNA	Blood	3/30 (10%)	'*Ca*. M. haemoalbiventris'	PR (FR)	No	Oliveira et al. (2021)
	qPCR (16S rRNA) cPCR (16S rRNA, 23S rRNA)	800 - 16S rRNA and 23S rRNA	Blood	8/42 (19%)	*Mycoplasma* sp. - phylogenetically related to 'Ca. M. haemodidelphis' and '*Ca*. M. haemoalbiventris'	PE/PI/BA/CE (FR)	No	Torres-Santos et al. (2024)
	qPCR (16S rDNA) cPCR (23S rRNA)	800 - 23S rRNA	Blood	9/10 (90%)	*Mycoplasma* sp.	RS (FR - road-killed animals)	-	Lignon et al. (2025)
Didelphis aurita	cPCR (16S rRNA)	740	Blood	22/30 (73.3%)	*Mycoplasma* sp. - phylogenetically related to '*Ca*. M. haemodidelphis'	MG (FR)	Yes	Orozco et al. (2022)
	cPCR (16S rRNA, 23S rRNA)	840 - 16S rRNA	Blood	3/15 (20%)	*Mycoplasma* sp. - phylogenetically related to 'Ca. M. haemodidelphis' (16S rRNA) and '*Ca*. M. haemoalbiventris' (23S rRNA)	RJ (FR)	No	Oliveira et al. (2023)
	cPCR (16S rDNA)	-	Liver	1/5 (20%)	No sequence	RJ/PR (FR)	No	Machado et al. (2024)
Didelphis marsupialis	cPCR (16S rRNA)	~600	Blood	7/45 (15.6%)	*Mycoplasma* sp. - phylogenetically related to 'Ca. M. haemodidelphis'	MA (FR)	No	Braga et al. (2023)

							and ‘*Ca*. M. haemoalbiventris’			
			cPCR (23S rRNA)	~800	Blood	2/16 (12,5%)	*Mycoplasma* sp. - phylogenetically related to ‘Ca. M. haemodidelphis’ and ‘*Ca*. M. haemoalbiventris’	PA/MA (FR)	No	Chagas-de-Souza et al. (2025)
		Monodelphis domestica	qPCR (16S rRNA) cPCR (16S rRNA, 23S rRNA)	800 - 16S rRNA	Blood	1/47 (2.1%)	*Mycoplasma* sp.	PE/PI/BA/CE (FR)	No	Torres-Santos et al. (2024)
Rodentia	Cricetidae	*Akodon* spp.	cPCR (16S rDNA)	~900	Liver	39/144 (27%)	*Mycoplasma* sp.	PR/RJ (FR)	No	Machado et al. (2024)
			cPCR (16S rRNA, RNaseP)	~1200 - 16S rRNA	Spleen	5/27 (18.5%)	*Mycoplasma* sp. - *M. haemofelis* group	RJ (FR)	No	Gonçalves et al. (2015)
		Akodon montensis	cPCR (16S rRNA, RNaseP)	-	Spleen	6/16 (37.5%)	No sequence	SP/SC (FR)	No	Gonçalves et al. (2015)
		Delomys dorsalis	cPCR (16S rRNA, RNaseP)	~1200 - 16S rRNA	Spleen	5/9 (55.5%)	*Mycoplasma* sp. - *M. haemofelis* group	RJ (FR)	No	Gonçalves et al. (2015)
		Oligoryzomys spp.	cPCR (16S rDNA)	~900	Liver	33/42 (78.5%)	*Mycoplasma* sp.	RJ/PR (FR)	No	Machado et al. (2024)
			cPCR (16S rRNA, RNaseP)	-	Spleen	3/11 (27.2%)	No sequence	TO/RJ/SP (FR)	No	Gonçalves et al. (2015)
		Oxymycterus sp.	cPCR (16S rDNA)	-	Liver	10/20 (40%)	No sequence	RJ/PR (FR)	No	Machado et al. (2024)

	cPCR (16S rRNA, RNaseP)	-	Spleen	2/3 (66.6%)	No sequence	RJ (FR)	No	Gonçalves et al. (2015)
Rhipidomys sp.	qPCR (16S rRNA) cPCR (16S rRNA, 23S rRNA)	-	Blood	3/6 (50%)	No sequence	PE/PI/BA/CE (FR)	No	Torres-Santos et al. (2024)
	cPCR (16S rRNA, RNaseP)	~1200 - 16S rRNA	Spleen	7/15 (46.6%)	*Mycoplasma* sp. - *M. haemofelis* group	GO/TO (FR)	No	Gonçalves et al. (2015)
Euryoryzomys russatus	cPCR (16S rDNA)	-	Liver	1/4 (25%)	No sequence	RJ/PR (FR)	No	Machado et al. (2024)
Sooretamys angouya	cPCR (16S rDNA)	-	Liver	1/12 (8.3%)	No sequence	RJ/PR (FR)	No	Machado et al. (2024)
Thaptomys nigrita	cPCR (16S rDNA)	-	Liver	1/2 (50%)	No sequence	RJ/PR (FR)	No	Machado et al. (2024)
Thrichomys apereoides	cPCR (16S rRNA, RNaseP)	~1200 - 16S rRNA	Spleen	1/10 (10%)	No sequence	RJ (FR)	No	Gonçalves et al. (2015)
Oligoryzomys nigripes	cPCR (16S rRNA, RNaseP)	~1200 - 16S rRNA	Spleen	7/24 (29.1%)	*Mycoplasma* sp. - *M. haemofelis* group	GO/RJ/MG/SP (FR)	No	Gonçalves et al. (2015)
Oligoryzomys flavescens	cPCR (16S rRNA, RNaseP)	-	Spleen	1/3 (33.3%)	No sequence	SP (FR)	No	Gonçalves et al. (2015)
Thrichomy fosteri	cPCR (16S rRNA, RNaseP)	~1200 - 16S rRNA	Spleen	4/18 (22.2%)	*Mycoplasma* sp. - *M. haemofelis* group	MS (FR)	No	Gonçalves et al. (2015)
Calomys tener	cPCR (16S rRNA, RNaseP)	-	Spleen	3/10 (30%)	No sequence	SP (FR)	No	Gonçalves et al. (2015)

Thrichomys sp.	cPCR (16S rRNA, RNaseP)	-	Spleen	2/6 (33.3%)	No sequence	BA/RN (FR)	No	Gonçalves et al. (2015)
Rhipidomys macrurus	cPCR (16S rRNA, RNaseP)	~1200 - 16S rRNA	Spleen	1/3 (33.3%)	*Mycoplasma* sp. - *M. haemofelis* group	PI (FR)	No	Gonçalves et al. (2015)
Oligoryzomys fornesi	cPCR (16S rRNA, RNaseP)	-	Spleen	1/2 (50%)	No sequence	GO (FR)	No	Gonçalves et al. (2015)
Nectomys squamipes	cPCR (16S rRNA, RNaseP)	-	Spleen	2/6 (33.3%)	No sequence	MG/MS (FR)	No	Gonçalves et al. (2015)
Nectomys rattus	cPCR (16S rRNA, RNaseP)	~1200 - 16S rRNA	Spleen	3/8 (37.5%)	*Mycoplasma* sp. - *M. haemofelis* group	MS/GO (FR)	No	Gonçalves et al. (2015)
Hylaeamys sp.	cPCR (16S rRNA, RNaseP)	-	Spleen	3/9 (33.3%)	No sequence	TO (FR)	No	Gonçalves et al. (2015)
Nectomys spp.	cPCR (16S rRNA, RNaseP)	~1200 - 16S rRNA	Spleen	1/5 (20%)	*Mycoplasma* sp. - *M. haemofelis* group	MS/GO (FR)	No	Gonçalves et al. (2015)
Necromys lasiurus	qPCR (16S rRNA) cPCR (16S rRNA, 23S rRNA)	-	Blood	2/5 (40%)	No sequence	PE/PI/BA/CE (FR)	No	Torres-Santos et al. (2024)
	cPCR (16S rRNA, RNaseP)	~1200 - 16S rRNA	Spleen	26/43 (60.4%)	*Mycoplasma* sp. - *M. haemofelis* group	RJ/MT/BA/MS/GO/TO (FR)	No	Gonçalves et al. (2015)
	cPCR (16S rDNA)	-	Liver	1/3 (33.3%)	No sequence	RJ/PR (FR)	No	Machado et al. (2024)

Muridae	*Rattus rattus**	cPCR (16S rRNA, RNaseP)	~1200 - 16S rRNA	Spleen	14/29 (48.2%)	*Mycoplasma* sp. - 99% identity to '*Ca*. M. haemomuris subsp. ratti'	GO/PA/MT/CE (FR)	No	Gonçalves et al. (2015)
		cPCR (16S rRNA)	1200	Blood	12/39 (30.7%)	*Mycoplasma* sp. phylogenetically related to '*Ca*. M. haemomuris'	MS (FR)	No	Gonçalves et al. (2020)
		qPCR (16S rRNA) cPCR (16S rRNA, 23S rRNA)	800 - 16S rRNA and 23S rRNA	Blood	2/9 (22.2%)	*Mycoplasma* spp. (99.65-100%)<br>99.89% identity '*Ca*. M. haemomuris subsp. ratti'	PE/PI/BA/CE (FR)	No	Torres-Santos et al. (2024)
		cPCR (16S rDNA)	-	Liver	2/2 (100%)	No sequence	RJ/PR (FR)	No	Machado et al. (2024)
	*Rattus norvegicus**	qPCR (16S rRNA) cPCR (16S rRNA)	1300		40/63 (63.5%)	*M. haemomuris* (98-100% identity)	PR (C)	No	Conrado et al. (2015)
	*Mus musculus**	cPCR (16S rRNA, RNaseP)	-	Spleen	1/14 (7.1%)	No sequence	SP/SC/PA/CE/TO (FR)	No	Gonçalves et al. (2015)
Erethizontidae	*Coendou spinosus*	cPCR (16S rRNA, 23S rRNA)	~900	Blood and spleen	2/9 (22%)	*Mycoplasma* sp. (*M. haemofelis* group)<br>~82% identity *M. haemocanis* and *M. haemofelis* (23S rRNA)	PR	No	Valente et al. (2020)
Caviidae	*Hydrochoerus hydrochaeris*	cPCR (16S rRNA)	~400	Blood	20/31 (64%)	*M. coccoides* (92% identity) and CMt (91% identity)	PR (C - 10 and FR - 21)	No	Vieira et al. (2009)

			cPCR (16S rRNA)	1200	Blood	7/14 (50%)	*Mycoplasma* sp.	MS (FR)	No	Gonçalves et al. (2020)
			cPCR (16S rRNA, 23S rRNA)	~800 - 16S rRNA and 23S rRNA	Blood	16/17 (94.1%)	*Mycoplasma* spp.<br><br>Novel - '*Ca*. M. haematohydrochaeris'	PR (FR)	No	Vieira et al. (2021)
			qPCR (16S rRNA) cPCR (23S rRNA)	800 - 23S rRNA	Blood	2/2 (100%)	*Mycoplasma* sp. phylogenetically related to '*Ca*. M. haematonasua'	RS (FR - road-killed animals)	-	Lignon et al. (2025)
		Kerodon rupestris	qPCR (16S rRNA) cPCR (16S rRNA, 23S rRNA)	800 - 16S rRNA and 23S rRNA	Blood	1/4 (25%)	*Mycoplasma* sp. - 98.13% identity '*Ca*. M. haematohydrochaerus'	PE/PI/BA/CE (FR)	No	Torres-Santos et al. (2024)
		Cavia aperea (guinea pig)	qPCR (16S rRNA) cPCR (23S rRNA)	800 - 23S rRNA	Blood	1/1 (100%)	*Mycoplasma* sp.	RS (FR - road-killed animals)	-	Lignon et al. (2025)
	Echimyidae	*Thrichomys laurentius*	qPCR (16S rRNA) cPCR (16S rRNA, 23S rRNA)	800 - 16S rRNA and 23S rRNA	Blood	15/118 (12.7%)	*Mycoplasma* sp.<br><br>84-92.97% identity '*Ca*. M. haemosphiggurus' (23S rRNA)	PE/PI/BA/CE (FR)	No	Torres-Santos et al. (2024)
			cPCR (16S rRNA, RNaseP)	~1200 - 16S rRNA	Spleen	2/17 (11.7%)	*Mycoplasma* sp. - *M. haemofelis* group	BA/CE/PI (FR)	No	Gonçalves et al. (2015)
Pilosa	Bradypodidae	*Bradypus tridactylus*	qPCR (16S rRNA) cPCR (16S rRNA, 23S rRNA)	~1400 - 16S rRNA	Blood and spleen	18/194 (9.3%)	*Mycoplasma* spp. - ~99% identity *M. wenyonii*	PA/RO (FR)	No	Oliveira et al. (2022)

	Bradypus sp.	qPCR (16S rRNA) cPCR (16S rRNA, 23S rRNA)	-	Blood and spleen	1/3 (33.3%)	No sequence	RO (FR)	No	Oliveira et al.(2022)
Choloepodidae	*Choloepus* sp.	qPCR (16S rRNA) cPCR (16S rRNA, 23S rRNA)	-	Blood and spleen	4/31 (12.9%)	*Mycoplasma* spp. (87.59% identity)	RO (FR)	No	Oliveira et al. (2022)
Myrmecophagidae	*Tamandua tetradactyla*	qPCR (16S rRNA) cPCR (16S rRNA, 23S rRNA)	~1400 - 16S rRNA<br>800 - 23S rRNA	Blood and spleen	8/40 (20%)	*Mycoplasma* spp. (~96% identity)<br>90% identity *M. haemomalae* - 23S rRNA	PA/RO/MS/SP/RS (FR)	No	Oliveira et al. (2022)
		cPCR (16S rRNA, 23S rRNA)	~500 - 16S rRNA	Blood and spleen	2/6 (33.3%)	*Mycoplasma* sp.	MS/SP (FR)	No	Sada et al. (2024)
	Myrmecophaga tridactyla	qPCR (16S rRNA) cPCR (16S rRNA, 23S rRNA)	-	Blood and spleen	12/67 (17.9%)	No sequence	MS/SP (FR)	No	Oliveira et al. (2022)
		cPCR (16S rRNA, 23S rRNA)	~500 - 16S rRNA	Blood and spleen	33/79 (41.7%)	*Mycoplasma* sp.	MS/SP (FR)	No	Sada et al. (2024)
Cyclopedidae	*Cyclopes didactylus*	cPCR (16S rRNA, 23S rRNA)	580 - 16S rRNA	Blood	4/9 (66.6%)	*Mycoplasma* sp.	MA/PE/PA/RN/AM (FR)	No	Rodrigues et al. (2025)
	Cyclopes thomasi	cPCR (16S rRNA, 23S rRNA)	-	Blood	1/1 (100%)	No sequence	AC (FR)	No	Rodrigues et al. (2025)
	Cyclopes rufus	cPCR (16S rRNA, 23S rRNA)	-	Blood	1/2 (50%)	No sequence	RO (FR)	No	Rodrigues et al. (2025)

Cingulata	Chlamyphoridae	*Euphractus sexcinctus*	qPCR (16S rRNA) cPCR (16S rRNA, 23S rRNA)	-	Blood and spleen	2/14 (14.2%)	No sequence	MS (FR)	No	Oliveira et al. (2022)
			cPCR (16S rRNA, 23S rRNA)	~600 - 16S rRNA<br>~400 - 23S rRNA	Blood and spleen	31/48 (64.6%)	*Mycoplasma* spp. phylogenetically related to '*Ca*. M. haematomaximus'	MS/SP (FR)	No	Sada et al. (2024)
			cPCR (16S rDNA, 23S rRNA)	800 - 23S rRNA	Blood	1/1 (100%)	*Mycoplasma* sp.	PR (FR - road-killed animals)	-	Lignon et al. (2025)
		Priodontes maximus	qPCR (16S rRNA) cPCR (16S rRNA, 23S rRNA)	~900 and 1400 - 16S rRNA	Blood and spleen	14/25 (56%)	*Mycoplasma* spp. (100% identity)	MS (FR)	No	Oliveira et al. (2022)
			cPCR (16S rRNA, 23S rRNA)	~500 - 16S rRNA	Blood and spleen	19/29 (65.5%)	*Mycoplasma* spp. phylogenetically related to '*Ca*. M. haematomaximus'<br>93.05% identity '*Ca*. M. haematominutum'	MS/SP (FR)	No	Sada et al. (2024)
	Dasypodidae	*Dasypus novemcinctus*	qPCR (16S rRNA) cPCR (16S rRNA, 23S rRNA)	-	Blood and spleen	1/14 (7.1%)	No sequence	RO/MS/SP (FR)	No	Oliveira et al. (2022)
					Blood and spleen	2/4 (50%)		MS/SP (FR)	No	Sada et al. (2024)
Cetartiodactyla	Iniidae	*Inia geoffrensis*	cPCR (16S rRNA)	1400	Blood	21/32 (65.6%)	*Mycoplasma* sp.	AM (FR)	No	Duarte-Benvenuto et al. (2022)

	Inia boliviensis	cPCR (16S rRNA)	1400	Blood	11/18 (61.1%)	*Mycoplasma* sp.	AM (FR)	No	Duarte-Benvenuto et al. (2022)
Pontoporiidae	*Pontoporia blainvillei (Franciscana dolphin)*	nPCR (16S rRNA) cPCR (23S rRNA)	330 and 1100 - 16S rRNA	Blood	3/42 (12.5%)	*Mycoplasma* sp.	SP (FR)	No	Duarte-Benvenuto et al. (2023)
Kogiidae	*Kogia breviceps (Pigmy sperm whale)*	qPCR (16S rRNA) nPCR (16S rRNA) cPCR (23S rRNA)	1100 - 16S rRNA	Blood	3/4 (75%)	*Mycoplasma* sp. 88.4-90.9% identity '*Ca*. Mycoplasma haemolamae' (23S rRNA)	SP (FR)	No	Duarte-Benvenuto et al. (2023)
	Kogia sima (Dwarf sperm whale)	qPCR (16S rRNA) nPCR (16S rRNA) cPCR (23S rRNA)	1100 - 16S rRNA	Blood	2/10 (20%)	*Mycoplasma* sp. 88.4-90.9% identity '*Ca*. Mycoplasma haemolamae' (23S rRNA)	SP (FR)	No	Duarte-Benvenuto et al. (2023)
Delphinidae	*Delphinus delphis (Common dolphin)*	qPCR (16S rRNA) nPCR (16S rRNA) cPCR (23S rRNA)	1100 - 16S rRNA	Blood	1/2 (50%)	*Mycoplasma* sp.	SP (FR)	No	Duarte-Benvenuto et al. (2023)
	Feresa attenuata (Pigmy killer whale)	qPCR (16S rRNA) nPCR (16S rRNA) cPCR (23S rRNA)	1100 - 16S rRNA	Blood	1/4 (25%)	*Mycoplasma* sp. 88.4-90.9% identity '*Ca*. Mycoplasma haemolamae' (23S rRNA)	SP (FR)	No	Duarte-Benvenuto et al. (2023)
	Orcinus orca (Killer whale)	qPCR (16S rRNA) nPCR (16S rRNA) cPCR (23S rRNA)	1100 - 16S rRNA	Blood	1/1 (100%)	*Mycoplasma* sp. 88.4-90.9% identity '*Ca*. Mycoplasma haemolamae' (23S rRNA)	SP (FR)	No	Duarte-Benvenuto et al. (2023)

		Sotalia guianensis (Guiana dolphin)	qPCR (16S rRNA) nPCR (16S rRNA) cPCR (23S rRNA)	330 - 16S rRNA	Blood	3/24 (12.5%)	*Mycoplasma* sp.	SP (FR)	No	Duarte-Benvenuto et al. (2023)
		Stenella coeruleoalba (Striped dolphin)	qPCR (16S rRNA) nPCR (16S rRNA) cPCR (23S rRNA)	-	Blood	1/2 (50%)	No sequence	SP (FR)	No	Duarte-Benvenuto et al. (2023)
		Stenella frontalis (Atlantic spotted dolphin)	qPCR (16S rRNA) nPCR (16S rRNA) cPCR (23S rRNA)	330 - 16S rRNA	Blood	2/7 (28.5%)	*Mycoplasma* sp.	SP (FR)	No	Duarte-Benvenuto et al. (2023)
		Steno bredanensis (Rough-toothed dolphin)	qPCR (16S rRNA) nPCR (16S rRNA) cPCR (23S rRNA)	1100 - 16S rRNA	Blood	1/4 (25%)	*Mycoplasma* sp.	SP (FR)	No	Duarte-Benvenuto et al. (2023)
	Suidae	*Sus scrofa**	qPCR (16S rRNA)	-	Blood and tissue	714 (50%)	*Mycoplasma suis*	SP (FR)	No	Dias et al. (2019)
			qPCR (16S rRNA)	-	Blood	38/65 (58.5%)	*Mycoplasma suis, Mycoplasma parvum*	GO (FR)	No	Fernandes et al. (2021)
			qPCR (16S rRNA) cPCR (23S rRNA)	800 - 23S rRNA	Blood	59/67 (88%)	*Mycoplasma* sp. - *M. suis* (99.64% identity) and *M. parvum* (88.78-99.86% identity)	SP (FR)	No	Santana et al. (2022)
	Cervidae	*Dama dama**	cPCR (16S rRNA)	-	Blood	1/1 (100%)	No sequence	MG (C)	No	Castillo et al. (2024)
Primates	Atelidae	*Alouatta spp.* (Howler monkeys)	cPCR (16S rRNA, RNaseP)	800 - 16S rRNA	Blood	18/68 (26.5%)	*Mycoplasma* sp. - closely related to '*Ca.*	SP (C)	Yes	Melo et al. (2019)

							M. kahanei' (*M. suis* group)			
		Alouatta caraya (Black howler monkey)	cPCR (16S rRNA)	~1000	Blood	9/14 (64.3%)	*Mycoplasma* sp. - closely related to '*Ca.* M. kahanei' (*M. suis* group)	PR (C and FR)	No	Cubilla et al. (2017a)
	Cebidae	*Sapajus apella* (Brown capuchin)	cPCR (16S rRNA, RNaseP)	~800 - 16S rRNA	Blood	29/65 (44.5%)	*Mycoplasma* sp. - 95-97% identity '*Ca.* M. haemomacaque'	MA (FR)	No	Bonato et al. (2015)
		Sapajus nigritus (Black-horned capuchin)	cPCR (16S rRNA)	-	Blood	1/24 (4.2%)	No sequence	PR (C and FR)	No	Cubilla et al. (2017a)
		Sapajus flavius (Marcgrave's capuchin)	cPCR (16S rRNA, 16S rDNA)	980 - 16S rDNA	Blood	8/12 (66.7%)	*Mycoplasma* sp - 95% identity '*Ca.* M. haemomacaque'	PB (C)	Yes	Ramalho et al. (2017)
		Saimiri sciureus (Squirrel monkey)	cPCR (16S rRNA, RNaseP)	~800 - 16S rRNA	Blood	4/16 (25%)	'*Ca.* M. kahanei' - *M. suis* group	MA (FR)	No	Bonato et al. (2015)
	Callitrichidae	*Saguinus midas niger* (Red-handed tamarin)	cPCR (16S rRNA, RNaseP)	-	Blood	2/7 (28.6%)	No sequence	MA (FR)	No	Bonato et al. (2015)
	Cercopithecidae	*Macaca mulatta (rhesus monkey)**	cPCR (16S rRNA, 23S rRNA)	535 - 16S rRNA	Blood	5/8 (62.5)<br>4/6 (66.7%)	*Mycoplasma* spp. - '*Ca*. M. haemomacaque' (*M. haemofelis* group)	RJ (C)	No	Mongruel et al. (2022a)
Chiroptera	Phyllostomidae	*Artibeus lituratus (Great Fruit-eating Bat)*	cPCR (16S rRNA, 23S rRNA)	~900 - 16S rRNA<br>~620 - 23S rRNA	Spleen	1/15 (6.6%)	*Mycoplasma sp. - M. haemofelis* group	PR (FR)	-	Collere et al. (2022)

	cPCR (16S rRNA, 23S rRNA, RNAseP)	~800 - 16S rRNA	Blood and spleen	11/37 (29.7%)	*Mycoplasma* sp. - *M. haemofelis* group	MS (FR)	-	Ikeda et al. (2022)
	cPCR (16S rRNA, 23S rRNA, RNAseP)	620 - 16S rRNA	Spleen	9/47 (33.3%)	*Mycoplasma* sp. - *M. haemofelis* group	AC (FR)	-	Silva et al. (2025)
Sturnira lilium	cPCR (16S rRNA, RNaseP)	-	Liver, spleen and heart	13/26 (50%)	No sequence	PR/PA (FR)	-	Ikeda et al. (2017)
Artibeus planirostris	cPCR (16S rRNA, RNaseP)	-	Liver and spleen	3/8 (37.5%)	No sequence	PA (FR)	-	Ikeda et al. (2017)
	cPCR (16S rRNA, 23S rRNA, RNaseP)	800 - 16S rRNA<br>~200 - 23S rRNA	Blood and spleen	17/33 (51.5%)	*Mycoplasma* sp. - *M. haemofelis* group	MS (FR)	-	Ikeda et al. (2022)
	cPCR (16S rRNA, 23S rRNA)	-	Spleen	10/54 (18.5%)	No sequence	AC (FR)	-	Silva et al. (2025)
Glossophaga soricina	cPCR (16S rRNA, RNaseP)	-	Whole blood	4/26 (15.3%)	No sequence	PA/TO/MT (FR - positive only TO)	-	Ikeda et al. (2017)
	cPCR (16S rRNA, 23S rRNA, RNaseP)	800 - 16S rRNA<br>~200 - 23S rRNA	Spleen	1/1 (100%)	*Mycoplasma* sp. - *M. haemofelis* group	MS (FR)	-	Ikeda et al. (2022)

	cPCR (16S rRNA, 23S rRNA)	-	Spleen	3/3 (100%)	No sequence	AC (FR)	-	Silva et al. (2025)
Platyrrhinus lineatus	cPCR (16S rRNA, 23S rRNA, RNaseP)	800 - 16S rRNA	Blood and spleen	5/23 (21.7%)	*Mycoplasma* sp. - *M. haemofelis* group	MS (FR)	-	Ikeda et al. (2022)
Carollia perspicillata	cPCR (16S rRNA, 23S rRNA, RNaseP)	-	Blood and spleen	3/5 (60%)	No sequence	MS (FR)	-	Ikeda et al. (2022)
	cPCR (16S rRNA, 23S rRNA)	620 - 16S rRNA<br>800 - 23S rRNA	Spleen	31/71 (43.6%)	*1Mycoplasma* sp. - *M. haemofelis* group<br>23S rRNA - 88.32% identity '*Ca.* M. haematomolossi'	AC (FR)	-	Silva et al. (2025)
Chiroderma villosum	cPCR (16S rRNA, 23S rRNA, RNaseP)	-	Spleen	1/1 (100%)	No sequence	MS (FR)	-	Ikeda et al. (2022)
Phyllostomus discolor	cPCR (16S rRNA, 23S rRNA, RNaseP)	800 - 16S rRNA	Blood and spleen	15/15 (100%)	*Mycoplasma* sp. - *M. haemofelis* group	MS (FR)	-	Ikeda et al. (2022)
	cPCR (16S rRNA, 23S rRNA)	620 - 16S rRNA	Spleen	4/4 (100%)	*Mycoplasma* sp. - *M. haemofelis* group	AC (FR)	-	Silva et al. (2025)
Anoura caudifer	cPCR (16S rRNA, 23S rRNA)	620 - 16S rRNA	Spleen	1/1 (100%)	*Mycoplasma* sp. - *M. haemofelis* group	AC (FR)	-	Silva et al. (2025)

	Carollia beikeith c.f.	cPCR (16S rRNA, 23S rRNA)	620 - 16S rRNA	Spleen	1/1 (100%)	*Mycoplasma* sp. - *M. haemofelis* group	AC (FR)	-	Silva et al. (2025)
	Dermanura cinereus	cPCR (16S rRNA, 23S rRNA)	620 - 16S rRNA	Spleen	2/2 (100%)	*Mycoplasma* sp. - *M. haemofelis* group	AC (FR)	-	Silva et al. (2025)
	Lophostoma silviculum	cPCR (16S rRNA, 23S rRNA)	620 - 16S rRNA	Spleen	2/2 (100%)	*Mycoplasma* sp. - *M. haemofelis* group	AC (FR)	-	Silva et al. (2025)
	Phyllostomus elongatus	cPCR (16S rRNA, 23S rRNA)	620 - 16S rRNA	Spleen	7/14 (50%)	*Mycoplasma* sp. - *M. haemofelis* group	AC (FR)	-	Silva et al. (2025)
	Phyllostomus hastatus	cPCR (16S rRNA, 23S rRNA)	620 - 16S rRNA	Spleen	6/6 (100%)	*Mycoplasma* sp. - *M. haemofelis* group	AC (FR)	-	Silva et al. (2025)
	Rhinophylla fischerae	cPCR (16S rRNA, 23S rRNA)	620 - 16S rRNA	Spleen	3/4 (75%)	*Mycoplasma* sp. - *M. haemofelis* group	AC (FR)	-	Silva et al. (2025)
	Stumira tildae	cPCR (16S rRNA, 23S rRNA)	620 - 16S rRNA	Spleen	1/2 (50%)	*Mycoplasma* sp. - *M. haemofelis* group	AC (FR)	-	Silva et al. (2025)
	Platyrhinus infuscus	cPCR (16S rRNA, 23S rRNA)	620 - 16S rRNA	Spleen	1/2 (50%)	*Mycoplasma* sp. - *M. haemofelis* group	AC (FR)	-	Silva et al. (2025)
Molossidae	*Molossus molossus*	cPCR (16S rRNA, RNaseP)	~800	Liver, spleen, heart	20/30 (66.6%)	*Mycoplasma sp.* - 93-96% identity *M. coccoides*	PA/PR/SP (FR)	-	Ikeda et al. (2017)
		cPCR (16S rRNA, 23S rRNA, RNaseP)	-	Blood	2/4 (50%)	No sequence	MS (FR)	-	Ikeda et al. (2022)

	Molossus rufus	cPCR (16S rRNA, RNaseP)	-	Whole blood	1/18 (5.5%)	No sequence	PA/MT (FR - positive only MT)	-	Ikeda et al. (2017)
	Eumops auripendulus	cPCR (16S rRNA, RNaseP)	-	Liver and spleen	1/1 (100%)	No sequence	PA (FR)	-	Ikeda et al. (2017)
	Molossus sp.	cPCR (16S rDNA)	700-900	Blood	1/2 (50%)	*Mycoplasma* sp.	PR (FR)	-	Santos et al. (2020)
Vespertilionidae	*Eptesicus spp.*	cPCR (16S rRNA, RNaseP)	-	Liver, spleen and heart	2/5 (40%)	No sequence	PR (FR)	-	Ikeda et al. (2017)
	Eptesicus furinalis	cPCR (16S rRNA, 23S rRNA, RNaseP)	800 - 16S rRNA	Spleen	3/9 (33.3%)	*Mycoplasma* sp. - *M. haemofelis* group	MS (FR)	-	Ikeda et al. (2022)
	Myotis nigricans	cPCR (16S rRNA, RNaseP)	-	Liver and spleen	1/1 (100%)	No sequence	PA (FR)	-	Ikeda et al. (2017)
Hematophagous	*Desmodus rotundus*	cPCR (16S rDNA)	700-900	Blood	5/6 (83.3%)	*Mycoplasma* sp.	PR (FR)	-	Santos et al. (2020)
		cPCR (16S rRNA, 23S rRNA, RNAseP)	~800 - 16S rRNA	Liver	11/12 (8.3%)	*Mycoplasma* sp. - *M. haemofelis* group	AM/ES/MT/PA/RS/SP/TO (FR)	-	Mello et al., (2023)
		cPCR (16S rRNA, 23S rRNA, RNAseP)	~1200 - 16S rRNA<br>~800 - 23S rRNA	Spleen	23/229 (10%)	*Mycoplasma* sp. - *M. haemofelis* group	PA/AM/RR (FR)	-	Mello et al. (2024)
	Diphylla ecaudata	cPCR (16S rDNA)	700-900	Blood	2/2 (100%)	*Mycoplasma* sp. - *M. haemofelis* group	PR (FR)	-	Santos et al. (2020)

			cPCR (16S rRNA, 23S rRNA, RNAseP)	~800 - 16S rRNA	Liver	1/12 (8.3%)	*Mycoplasma* sp. - *M. haemofelis* group	ES (FR)	-	Mello et al. (2023)

*Exotic animals in Brazil
